# Antimicrobial Investigation of Phthalimide and *N*-Phthaloylglycine Esters: Activity, Mechanism of Action, Synergism and Ecotoxicity

**DOI:** 10.3390/life15040518

**Published:** 2025-03-21

**Authors:** Francinara S. Alves, Abraão P. Sousa, Alexandre Almeida-Júnior, Priscila S. V. Lima, Marcelo F. R. Silva, José L. F. M. Galvão, Edeltrudes O. Lima, Helivaldo D. S. Souza, José A. S. Luis, Petrônio F. Athayde-Filho, Gabriela F. Fiss

**Affiliations:** 1 Laboratório de Pesquisa em Bioenergia e Síntese Orgânica (LPBS), Department of Chemistry, Federal University of Paraíba, João Pessoa 58051-900, Brazil; francinaraufpb@gmail.com (F.S.A.); abraaoiqmpinheiro@gmail.com (A.P.S.); alexandre_junior.02@hotmail.com (A.A.-J.); helivaldog3@gmail.com (H.D.S.S.); athayde-filho@quimica.ufpb.br (P.F.A.-F.); 2 Núcleo de Química de Heterociclos (NUQUIMHE), Department of Chemistry, Federal University of Santa Maria, Santa Maria 97105-900, Brazil; priscila.svl@hotmail.com; 3 Laboratório Multiusuário de Caracterização e Análises (LMCA), Institute of Research in Drugs and Medicines, Federal University of Paraíba, João Pessoa 58051-900, Brazil; marcelo.mfrs@ltf.ufpb.br; 4 Nova Esperança University Hospital, Nova Esperança College, João Pessoa 58067-695, Brazil; luksfmgalvao_4@hotmail.com; 5 Department of Pharmaceutical Sciences, Federal University of Paraíba, João Pessoa 58051-900, Brazil; edelolima@yahoo.com.br; 6 Center of Education and Health, Federal University of Campina Grande, Cuité 58429-600, Brazil; jose.alixandre@professor.ufcg.edu.br

**Keywords:** antimicrobial activity, *Artemia salina*, *Candida albicans*, chloramphenicol, docking molecular, ergosterol, fluconazole, *Pseudomonas aeruginosa*, *Staphylococcus aureus*

## Abstract

Motivated by the search for novel antimicrobials against opportunistic resistant pathogens and based on the reported antimicrobial activity of phthalimides, two series of phthalimide and *N*-phthaloylglycine esters were designed to investigate whether the addition of butyl and aryl groups enhances their antimicrobial properties. Thus, in vitro antimicrobial activity, antifungal mechanism of action, effect combined with Chloramphenicol, in silico/vitro toxicity, and a docking molecular were studied. Phthalimide and *N*-phthaloylglycine aryl esters were obtained in yields of 75–98%. Phthalimide aryl ester **3b** (R = Me) showed the best results against Gram-(+) and Gram-(−) bacteria, *S. aureus* and *P. aeruginosa*, respectively, and yeast fungi, *C. tropicalis* and *C. albicans*, with MIC values equal to 128 µg·mL^−1^. Regarding the antifungal mechanism of action on *C. albicans*, the MIC values of compound **3b** changed from 128 to 1024 µg·mL^−1^ in the presence of ergosterol. Furthermore, compound **3b** showed synergy with Chloramphenicol against *P. aeruginosa*, with a FICI value equal to 0.5. Finally, the four most promising compounds had their in silico/vitro toxicity evaluated, which showed moderate toxicity to non-toxicity on *Artemia salina* larvae. With the exception of Chloramphenicol, all selected compounds, including Fluconazole, are potentially hepatotoxic, but they were predicted not to cause skin sensitization, suggesting a potential application for topical use. Molecular docking revealed that compound **3b** exhibits superior binding affinity and stability with the 50S ribosomal subunit (−92.69 kcal·mol^−1^) compared to Chloramphenicol, and a unique π–sulfur interaction with CYP51, suggesting its potential as a dual-action antibacterial and antifungal candidate against resistant pathogens.

## 1. Introduction

It is estimated that the number of deaths per year related to resistant superbugs to traditional drugs will increase from 700 thousand to 1.2 million, warns the World Health Organization (WHO) in its report “Incentivising the development of new antibacterial treatments 2023” [1]. Considering that pharmaceutical companies have abandoned research into novel antibiotics, this report alerts the scientific community to the socioeconomic crisis posed by antimicrobial resistance, which causes more deaths than HIV or malaria. Just enough antibiotic for the necessary time needs to be prescribed to kill all bacteria in a diseased organism. When administered inappropriately, antibiotics kill only the most sensitive bacteria, and those that survive gain more resistance when exposed again to the same antibiotic [2].

Most antibiotics on the market are variations of drugs developed in the 1980s. According to the WHO report, only 77 novel treatments are in clinical development worldwide and are unlikely to reach the market. According to the organization, there is no viable market for novel antibiotics because the financial return does not cover the costs of their investment. It will be up to academia to finance new research. The goal is to place four novel antibiotics on the market by 2030 [1].

WHO priority-resistant microbial pathogens are especially dangerous because some are opportunistic, such as *Candida albicans* [3], a critical priority group [4], and *Pseudomonas aeruginosa* and *Staphylococcus aureus* [5,6]. Opportunistic infections occur when fungi, bacteria, viruses, or parasites damage immunocompromised patients, or when infections become persistently localized and/or disseminated [7]. Three main classes of antifungals generally treat candidiasis, an infection most commonly caused by *C. albicans*, polyenes, echinocandins, and azoles. Among azoles, Fluconazole is the most used due to its high effectiveness and low cost. However, recent studies have shown increased resistance of *C. albicans* to Fluconazole [8].

**Figure 1:**
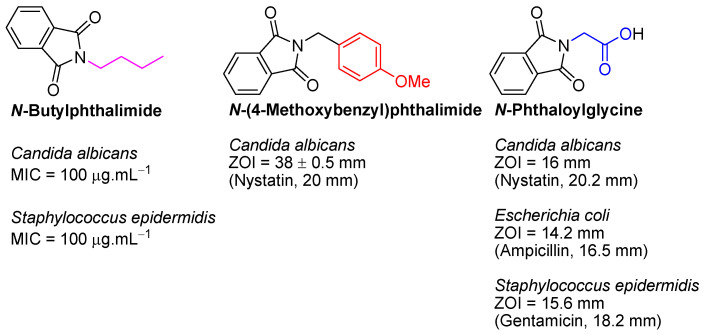
Best antimicrobial results for *N*-butylphthalimide [9], *N*-(4-methoxybenzyl)phthalimide [10], and *N*-phthaloylglycine [11]. MIC: Minimum Inhibitory Concentration; ZOI: Zone of Inhibition.

In this sense, the antimicrobial effects of *N*-substituted phthalimides have been investigated, especially against *C. albicans* [9,10]. Furthermore, *N*-phthaloyl amino acids have also demonstrated antimicrobial activity [11] (Figure 1). Phthalimide derivatives can also be found in commercial drugs such as Apremilast [12] and Pomalidomide [13], which are used in the treatment of psoriasis and multiple myeloma, respectively. Nevertheless, perhaps the best-known drugs with a phthalimide nucleus are (*R*) and (*S*)-thalidomide, famous for their sedative–hypnotic activity and tragic teratogenic activity, respectively. Nowadays, thalidomide is used for the treatment of leprosy, caused by infection with *Mycobacterium leprae*, as well as for multiple myeloma [14].

Our research group has been studying the antimicrobial properties of 2-phthalylethaneselenoate as a potential fungicidal against *Cryptococcus gatti*, with a Minimum Fungicidal Concentration (MFC) of 2.5 µg·mL^−1^ (Fluconazole, MFC = 2 µg·mL^−1^) [15], and *N*-phthaloylglycine alkyl esters, mainly against *Candida* species, whose best result showed a Minimum Inhibitory Concentration (MIC) of 64 µg·mL^−1^ against *C. tropicalis* (Amphotericin B, MIC = 100 µg·mL^−1^) [16]. Now, we are engaged in investigating the antimicrobial effects of phthalimide and *N*-phthaloylglycine aryl esters, from the determination of MIC values to the antifungal mechanism of action, and the combined effect with Chloramphenicol. Based on the reported antimicrobial activity of phthalimides [9,10,11], we want to verify whether adding butyl and aryl groups to phthalimide or *N*-phthaloylglycine improves the antimicrobial activity. Furthermore, for the most promising compounds, we intend to evaluate ecotoxicity on *Artemia salina* larvae as a possible indication of aquatic pollution [17], as well as conduct a molecular docking study to understand the mechanism of action at the molecular level.

## 2. Materials and Methods

### 2.1. Chemistry

All common reagents were purchased from commercial suppliers and used without further purification. Compounds were synthesized at LPBS-UFPB, and melting points were measured using QUIMIS equipment, model Q340S23 (Diadema, Brazil). ^1^H and ^13^C Nuclear Magnetic Resonance (NMR) spectra were acquired at LMCA-UFPB, using Bruker Ascend (Coventry, UK) and Bruker (Billerica, MA, USA) spectrometers at 400 and 500 MHz, respectively, for ^1^H, at 298 K, and 5 mm tubes. Infrared (IR) spectra were acquired on a Shimadzu spectrometer (Kyoto, Japan), model IRPrestige-21, at Laboratório de Síntese Orgânica Medicinal (LASOM-UFPB), using KBr pellets, and Núcleo de Pesquisa e Extensão de Combustíveis e de Materiais (NPE-LACOM-UFPB), using KBr pellets and attenuated total reflection (ATR) technique. High-Resolution Mass Spectrometry (HRMS) was performed at LMCA-UFPB, using a Shimadzu HPLC (Kyoto, Japan) coupled to a Bruker MicroTOF II (Billerica, MA, USA) with an electrospray ion (ESI) source, and reported as *m*/*z* (relative intensity) for molecular ion [M + Na]^+^; Acquisition Parameters: Ion Polarity Positive, Capillary 4500 V, End Plate Offset −500 V, Nebulizer 4.0 Bar, Dry Heater 200 °C, Dry Gas 8.0 L·min^−1^, Divert Valve Waste. Antimicrobial assays were performed at Laboratório de Atividades Antibacteriana e Antifúngica de Produtos Naturais e/ou Sintéticos Bioativos-UFPB. Ecotoxicity tests were performed at LPBS-UFPB.

#### 2.1.1. General Procedure for Synthesis of Potassium Salts of Phthalimide and *N*-Phthaloylglycine

An ethanolic solution (40 mL) of potassium hydroxide (20 mmol) was slowly added to a hot ethanolic solution (40 mL) of phthalimide or *N*-phthaloylglycine (20 mmol). The reactional mixture was maintained under magnetic stirring at room temperature for 2 h. Afterward, the precipitate was filtered under reduced pressure and washed with excess cold ethanol. Potassium salts of phthalimide (80% yield) and *N*-phthaloylglycine (96% yield) were obtained as white solids with m.p. > 300 °C.

#### 2.1.2. General Procedure for Synthesis of Esters (**2a**–**d**)

**Scheme 1:**
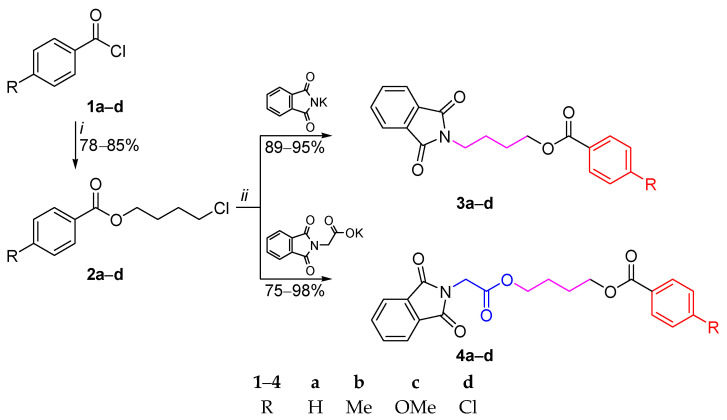
Reagents and reactional conditions to obtain esters (**2a**–**d**): (*i*) THF, 1 mol% ZnCl_2_, 65 °C (reflux), 2 h; Reagents and reactional conditions to obtain phthalimide and *N*-phthaloylglycine esters (**3a**–**d** and **4a**–**d**): (*ii*) 1 mol% NaI, DMF, 100 °C, 20–24 h.

Esters (**2a**–**d**) were synthesized according to a method previously reported [18,19], with some adjustments. A mixture of aroyl chloride **1** (10 mmol), THF (10 mL), and 1 mol% ZnCl_2_ (0.1 mmol) was maintained under magnetic stirring at 65 °C for 2 h (Scheme 1). Afterward, the reactional mixture was cooled to room temperature and extracted with dichloromethane (20 mL), washed with ice water (3 × 20 mL), and saturated sodium bicarbonate solution (3 × 20 mL). Then, the organic layer was dried with anhydrous sodium sulfate, filtered, and the solvent was evaporated under reduced pressure. Esters (**2a**–**d**) were purified by chromatographic column from hexane:ethyl acetate 97:3 and obtained in yields of 78–85%.

- 4-Chlorobutyl benzoate (**2a**) in 85% yield; colorless liquid; full data were found to be identical with the ones described in Ref. [19].
- 4-Chlorobutyl 4-methylbenzoate (**2b**) in 81% yield; colorless liquid; full data were found to be identical with the ones described in Ref. [19].
- 4-Chlorobutyl 4-methoxybenzoate (**2c**) in 80% yield; yellow liquid; full data were found to be identical with the ones described in Ref. [18].
- 4-Chlorobutyl 4-chlorobenzoate (**2d**) in 78% yield; yellow liquid; full data were found to be identical with the ones described in Ref. [19].

#### 2.1.3. General Procedure for Synthesis of Phthalimide and *N*-Phthaloylglycine Esters (**3a**–**d** and **4a**–**d**)

A mixture of ester **2** (1 mmol), 1 mol% NaI (0.01 mmol), anhydrous DMF (2 mL), and potassium salt of phthalimide or *N*-phthaloylglycine (1.4 mmol) was stirred at 100 °C for 20–24 h (Scheme 1), which was monitored by thin-layer chromatography (TLC). Afterward, the reactional mixture was cooled to room temperature and extracted with ethyl acetate (10 mL), washed with water (3 × 10 mL), and saturated sodium chloride solution (3 × 10 mL). Then, the organic layer was dried with anhydrous sodium sulfate, filtered, and the solvent was evaporated under reduced pressure. Phthalimide and *N*-phthaloylglycine esters (**3a**–**d** and **4a**–**d**) were purified by crystallization from ethanol:water 4:1 and obtained in yields of 75–98%. Full NMR, IR, and HRMS spectra for phthalimide and *N*-phthaloylglycine esters (**3a**–**d** and **4a**–**d**) are available in Figures S1–S32.

- 4-(1,3-Dioxoisoindolin-2-yl)butyl benzoate (**3a**, C_19_H_17_NO_4_) in 91% yield; *R*_f_: 0.43 (hexane:ethyl acetate 7:3); white solid; m.p.: 98–100 °C (Ref. [20] 97–98 °C); ^1^H NMR (400 MHz, CDCl_3_): *δ* = 8.02 (dd, *J* = 8.1, 1.0 Hz, 2H, H_Ar_), 7.84 (dd, *J* = 5.5, 3.1 Hz, 2H, H_Ar_), 7.72–7.69 (m, 2H, H_Ar_), 7.54 (t, *J* = 7.5 Hz, 1H, H_Ar_), 7.42 (t, *J* = 7.6 Hz, 2H, H_Ar_), 4.35 (t, *J* = 6.8 Hz, 2H, CH_2_), 3.77 (t, *J* = 6.8 Hz, 2H, CH_2_), 1.87–1.84 (m, 4H, 2 × CH_2_) ppm; ^13^C NMR (100 MHz, CDCl_3_): *δ* = 168.5, 166.6 (2 × C=O), 134.4, 134.0, 133.0, 132.2, 130.3, 129.6, 128.4, 123.7, 123.3 (9 × C_Ar_), 64.4, 37.7, 26.3, 25.4 (4 × CH_2_) ppm; IR (KBr): *ν* = 3068 (H_Ar_), 2965, 2938, 2868 (H_alkanic_), 1771, 1705 (C=O), 1602, 1462 (C=C), 1273, 1105, 1040 (C–O/N), 868, 705 (Ar) cm^−1^; HRMS (ESI): calcd for C_19_H_17_NNaO_4_ ([M + Na]^+^) 346.1050, found 346.1052.
- 4-(1,3-Dioxoisoindolin-2-yl)butyl 4-methylbenzoate (**3b**, C_20_H_19_NO_4_) in 89% yield; *R*_f_: 0.48 (hexane:ethyl acetate 7:3); white solid; m.p.: 121–122 °C (Ref. [21] yellow liquid); ^1^H NMR (500 MHz, CDCl_3_): *δ* = 7.91 (d, *J* = 8.4 Hz, 2H, H_Ar_), 7.84 (dd, *J* = 5.3, 3.1 Hz, 2H, H_Ar_), 7.71 (dd, *J* = 5.5, 3.1 Hz, 2H, H_Ar_), 7.21 (d, *J* = 8.1 Hz, 2H, H_Ar_), 4.33 (t, *J* = 6.8 Hz, 2H, CH_2_), 3.77 (t, *J* = 6.8 Hz, 2H, CH_2_), 2.39 (s, 3H, CH_3_), 1.87–1.81 (m, 4H, 2 × CH_2_) ppm; ^13^C NMR (125 MHz, CDCl_3_): *δ* = 168.5, 166.7 (2 × C=O), 143.6, 134.0, 132.2, 129.7, 129.1, 127.6 (6 × C_Ar_), 64.2, 37.7, 26.3, 25.5 (4 × CH_2_), 21.7 (CH_3_) ppm; IR (KBr): *ν* = 3062 (H_Ar_), 2968, 2919, 2871 (H_alkanic_), 1770, 1710 (C=O), 1607, 1470 (C=C), 1280, 1122, 1059 (C–O/N), 755, 726 (Ar) cm^−1^; HRMS (ESI): calcd for C_20_H_19_NNaO_4_ ([M + Na]^+^) 360.1205, found 360.1206.
- 4-(1,3-Dioxoisoindolin-2-yl)butyl 4-methoxybenzoate (**3c**, C_20_H_19_NO_5_) in 93% yield; *R*_f_: 0.48 (hexane:ethyl acetate 7:3); white solid; m.p.: 110–112 °C; ^1^H NMR (500 MHz, CDCl_3_): *δ* = 7.97 (d, *J* = 6.9 Hz, 2H, H_Ar_), 7.83 (d, 2H, *J* = 8.4 Hz, H_Ar_), 7.71 (d, *J* = 3.1 Hz, 2H, H_Ar_), 6.89 (d, *J* = 8.9 Hz, 2H, H_Ar_), 4.31 (t, *J* = 6.1 Hz, 2H, CH_2_), 3.84 (s, 3H, CH_3_) 3.76 (t, *J* = 6.9 Hz, 2H, CH_2_), 1.85–1.83 (m, 4H, 2 × CH_2_) ppm; ^13^C NMR (125 MHz, CDCl_3_): *δ* = 168.5, 166.4 (2 × C=O), 163.4, 134.0, 132.2, 131.6, 123.3, 122.8, 113.6 (7 × C_Ar_), 64.1 (CH_2_), 55.5 (CH_3_), 37.7 (CH_2_), 26.3 (CH_2_), 25.5 (CH_2_) ppm; IR (KBr): *ν* = 3019 (H_Ar_), 2980, 2923, 2896 (H_alkanic_), 1773, 1701 (C=O), 1606, 1511 (C=C), 1253, 1161, 1127, 1055 (C–O/N), 852, 720 (Ar) cm^−1^; HRMS (ESI): calcd for C_20_H_19_NNaO_5_ ([M + Na]^+^) 376.1155, found 376.1157.
- 4-(1,3-Dioxoisoindolin-2-yl)butyl 4-chlorobenzoate (**3d**, C_19_H_16_ClNO_4_) in 95% yield; *R*_f_: 0.42 (hexane:ethyl acetate 7:3); white solid; m.p.: 98 °C; ^1^H NMR (400 MHz, CDCl_3_): *δ* = 7.95 (d, *J* = 8.8 Hz, 2H, H_Ar_), 7.84 (dd, *J* = 5.5, 3.1 Hz, 2H, H_Ar_), 7.71 (dd, *J* = 5.4, 3.1 Hz, 2H, H_Ar_), 7.39 (d, *J* = 8.8 Hz, 2H, H_Ar_), 4.34 (t, *J* = 6.1 Hz, 2H, CH_2_), 3.76 (t, 2H, *J* = 6.1 Hz, CH_2_), 1.84–1.83 (m, 4H, 2 × CH_2_) ppm; ^13^C NMR (100 MHz, CDCl_3_): *δ* = 168.5, 165.8 (2 × C=O), 139.4, 134.1, 132.2, 131.1, 128.8, 123.3 (6 × C_Ar_), 64.6, 37.6, 26.2, 25.4 (4 × CH_2_) ppm; IR (KBr): *ν* = 3064 (H_Ar_), 2968, 2923, 2868 (H_alkanic_), 1773, 1713 (C=O), 1607, 1462 (C=C), 1285, 1122, 1055 (C–O/N), 759, 722 (Ar) cm^−1^; HRMS (ESI): calcd for C_19_H_16_ClNNaO_4_ ([M + Na]^+^) 380.0660, found 380.0662.
- 4-(2-(1,3-Dioxoisoindolin-2-yl)acetoxy)butyl benzoate (**4a**, C_21_H_19_NO_6_) in 83% yield; *R*_f_: 0.43 (hexane:ethyl acetate 2:1); white solid; m.p.: 59–60 °C; ^1^H NMR (500 MHz, CDCl_3_): *δ* = 8.02 (d, *J* = 7.1 Hz, 2H, H_Ar_), 7.88 (dd, *J* = 5.4, 3.0 Hz, 2H, H_Ar_), 7.73 (dd, *J* = 5.5, 3.0 Hz, 2H, H_Ar_), 7.56 (t, *J* = 7.4 Hz, 1H, H_Ar_), 7.44 (t, *J* = 7.7 Hz, 2H, H_Ar_), 4.45 (s, 2H, CH_2_), 4.33 (t, *J* = 5.9 Hz, 2H, CH_2_), 4.25 (t, *J* = 5.9 Hz, 2H, CH_2_), 1.85–1.81 (m, 4H, CH_2_) ppm; ^13^C NMR (125 MHz, CDCl_3_): *δ* = 167.6, 167.4, 166.6 (3 × C=O), 134.3, 133.0, 132.1, 129.6, 128.5, 123.7 (6 × C_Ar_), 65.4, 64.4, 39.0, 25.4, 25.4 (5 × CH_2_) ppm; IR (ATR): *ν* = 3053, 3030 (H_Ar_), 2964, 2943, 2897 (H_alkanic_), 1770, 1743, 1705 (C=O), 1600, 1467 (C=C), 1263, 1247, 1193, 1111, 1072, 1039 (C–O/N), 798 (Ar) cm^−1^; HRMS (ESI): calcd for C_21_H_19_NNaO_6_ ([M + Na]^+^) 404.1105, found 404.1100.
- 4-(2-(1,3-Dioxoisoindolin-2-yl)acetoxy)butyl 4-methylbenzoate (**4b**, C_22_H_21_NO_6_) in 75% yield; *R*_f_: 0.41 (hexane:ethyl acetate 2:1); beige solid; m.p.: 100–101 °C; ^1^H NMR (400 MHz, CDCl_3_): *δ* = 7.91 (d, *J* = 8.3 Hz, 2H, H_Ar_), 7.88 (dd, *J* = 5.5, 3.1 Hz, 2H, H_Ar_), 7.73 (dd, *J* = 5.5, 3.1 Hz, 2H, H_Ar_), 7.23 (d, *J* = 8.3 Hz, 2H, H_Ar_), 4.45 (s, 2H, CH_2_), 4.31 (t, *J* = 6.0 Hz, 2H, CH_2_), 4.24 (t, *J* = 6.0 Hz, 2H, CH_2_), 2.40 (s, 3H, CH_3_), 1.83–1.80 (m, 4H, CH_2_) ppm; ^13^C NMR (100 MHz, CDCl_3_): *δ* = 167.5, 167.3, 166.7 (3 × C=O), 143.7, 134.3, 132.1, 129.7, 129.2, 127.6, 123.7 (7 × C_Ar_), 65.5, 64.2, 39.0, 25.4, 25.4 (5 × CH_2_), 21.8 (CH_3_) ppm; IR (KBr): *ν* = 3104 (H_Ar_), 2953, 2934, 2853 (H_alkanic_), 1755, 1713 (C=O), 1607, 1417 (C=C), 1288, 1207, 1114, (C–O/N), 759, 719 (Ar) cm^−1^; HRMS (ESI): calcd for C_21_H_19_NNaO_6_ ([M + Na]^+^) 418.1261, found 418.1242.
- 4-(2-(1,3-Dioxoisoindolin-2-yl)acetoxy)butyl 4-methoxybenzoate (**4c**, C_22_H_21_NO_7_) in 98% yield; *R*_f_: 0.40 (hexane:ethyl acetate 2:1); white solid; m.p.: 98 °C; ^1^H NMR (400 MHz, CDCl_3_): *δ* = 7,98–7.96 (m, 2H, H_Ar_), 7.88 (dd, *J* = 5.5, 3.0 Hz, 2H, H_Ar_), 7.73 (dd, *J* = 5.5, 3.1 Hz, 2H, H_Ar_), 6.93–6.90 (m, 2H, H_Ar_), 4.45 (s, 2H, CH_2_), 4.30 (t, *J* = 5.5 Hz, 2H, CH_2_), 4.24 (t, *J* = 4.1 Hz, 2H, CH_2_), 3.86 (s, 3H, CH_3_), 1.81 (m, 4H, CH_2_) ppm; ^13^C NMR (100 MHz, CDCl_3_): *δ* = 167.6, 167.4, 166.4 (3 × C=O), 163.4, 134.3, 132.1, 131.7, 123.7, 122.7, 113.7 (7 × C_Ar_), 65.5 (CH_2_), 64.1 (CH_2_), 55.5 (CH_3_), 39.0 (CH_2_), 25.4 (CH_2_) ppm; IR (KBr): *ν* = 2956, 2837 (H_alkanic_), 1771, 1721 (C=O), 1607, 1509 (C=C), 1262, 1231, 1162, 1121, 1029 (C–O/N), 771, 709 (Ar) cm^−1^; HRMS (ESI): calcd for C_22_H_21_NNaO_7_ ([M + Na]^+^) 434.1210, found 434.1193.
- 4-(2-(1,3-Dioxoisoindolin-2-yl)acetoxy)butyl 4-chlorobenzoate (**4d**, C_21_H_18_ClNO_6_) in 85% yield; *R*_f_: 0.43 (hexane:ethyl acetate 2:1); white solid; m.p.: 74–77 °C; ^1^H NMR (400 MHz, CDCl_3_): *δ* = 7.97–7.95 (m, 2H, H_Ar_), 7.88 (dd, *J* = 5.5, 3.0 Hz, 2H, H_Ar_), 7.74 (dd, *J* = 5.5, 3.0 Hz, 2H, H_Ar_), 7.43–7.40 (m, 2H, H_Ar_), 4.46 (s, 2H, CH_2_), 4.33 (t, *J* = 6.1 Hz, 2H, CH_2_), 4.25 (t, *J* = 6.1 Hz, 2H, CH_2_), 1.83 (m, 4H, CH_2_) ppm; ^13^C NMR (100 MHz, CDCl_3_): *δ* = 167.4, 167.2, 165.6 (3 × C=O), 139.4, 134.2, 132.0, 130.9, 128.7, 128.6, 123.6 (7 × C_Ar_), 65.3, 64.5, 38.9, 25.3, 25.2 (5 × CH_2_) ppm; IR (ATR): *ν* = 3082, 3047 (H_Ar_), 2974, 2951, 2891 (H_alkanic_), 1743, 1710 (C=O), 1591, 1467 (C=C), 1294, 1193, 1112, 1087, 1016 (C–O/N), 858, 798, 756, 732, 686 (Ar) cm^−1^; HRMS (ESI): calcd for C_21_H_18_ClNNaO_6_ ([M + Na]^+^) 438.0715, found 438.0718.

### 2.2. In Silico/Vitro Antimicrobial Evaluation

In silico antimicrobial activity for phthalimide and *N*-phthaloylglycine esters (**3a**–**d** and **4a**–**d**) against *Candida albicans* was predicted through the open-access electronic site MolPredictX “https://www.molpredictx.ufpb.br (2 January 2025)” [22].

In vitro antimicrobial activity of phthalimide and *N*-phthaloylglycine esters (**3a**–**d** and **4a**–**d**) against microorganisms from American Type Culture Collection (ATCC) and Laboratório de Micologia (LM)—Gram-(+) (*Staphylococcus aureus* ATCC 25923 and LM 01) and Gram-(−) bacteria (*Escherichia coli* ATCC 25922, and *Pseudomonas aeruginosa* ATCC 25853), yeast (*C. albicans* ATCC 76485 and LM 65, and *C. tropicalis* ATCC 13803 and LM 303), and filamentous fungi (*Aspergillus flavus* ATCC 4603, and *A. niger* ATCC 6275)—was evaluated using broth microdilution method to determine MIC values, according to Clinical and Laboratory Standards Institute (CLSI), documents M07-A10 [23] and M27-A3 [24]. Chloramphenicol and Fluconazole were used as antimicrobial controls against bacteria and fungi, respectively. All experiments were performed in triplicate.

### 2.3. Antifungal Mechanism of Action

The antifungal mechanism of action of compound **3b** on strains of *C. albicans* ATCC 76485 was investigated by sorbitol and ergosterol assays [25,26].

The determination of MIC values of compound **3b** in the presence of sorbitol or ergosterol was carried out using the broth microdilution method. In this assay, 100 µL of Sabouraud Dextrose Broth (SDB), supplemented with sorbitol (0.8 M) or ergosterol (400 µg·mL^−1^), was distributed into 96 “U”-shaped wells of a microtiter plate. Next, 100 µL of serial concentrations (1024, 512, 256, 128, 64, 32, 16, 8, 4, 2, 1, and 0.5 µg·mL^−1^) of compound **3b** was added to the plate. Finally, 10 µL of fungal suspension was added to the plate. At the same time, fungal viability controls (100 µL of SDB with sorbitol or ergosterol, and 100 µL of sterile distilled water with 10 µL of fungal suspension) were conducted. Caspofungin and fluconazole were used as positive controls. Assays were incubated at 35 ± 2 °C for 24–48 h. All experiments were performed in duplicate.

### 2.4. Synergy Checkerboard Assay

The combined effect of compound **3b** with Chloramphenicol was studied on strains of *S. aureus* ATCC 25923 and *P. aeruginosa* ATCC 25853.

In this assay, 100 µL of SDB was distributed into 96 “U”-shaped wells of a microtiter plate. Next, 50 µL of established concentrations (MIC × 8, MIC × 4, MIC × 2, MIC, MIC ÷ 2, MIC ÷ 4, MIC ÷ 8) of compound **3b** (A) were added in a vertical direction, and 50 µL of established concentrations of Chloramphenicol (B) were added in a horizontal direction. Finally, 10 µL of bacterial suspension was added, previously adjusted to a tube of 0.5 McFarland standard [23,24]. Assays were incubated at 35 ± 2 °C for 3–5 days. Then, after incubation, Fractional Inhibitory Concentration (FIC) was calculated, where FIC_A_ = MIC_A+B_/MIC_A_ and FIC_B_ = MIC_B+A_/MIC_B_. Next, Fractional Inhibitory Concentration Index (FICI) was calculated, where FICI = FIC_A_ + FIC_B_ [27,28].

### 2.5. In Silico/Vitro Toxicity Evaluation

In silico toxicity and lipophilicity for phthalimide and *N*-phthaloylglycine esters (**3b**, **3c**, **4a**, **4c**) were predicted through the open-access electronic site pkCSM “https://biosig.lab.uq.edu.au/pkcsm/prediction (2 January 2025)” [29].

In vitro ecotoxicity of phthalimide and *N*-phthaloylglycine esters (**3b**, **3c**, **4a**, **4c**) on *Artemia salina* larvae was evaluated according to the adapted method reported by Sousa et al. [30], with some adjustments.

In a rectangular aquarium of 3 L capacity and using 20 W light at a height of 20 cm from the water surface, 1 g of *A. salina* cysts was placed in 1 L of saline solution (30 g.L^−1^, pH ~ 8) and aerated at 27 °C for 48 h. The toxicity was evaluated at concentrations of 125, 250, and 375 µg·mL^−1^. To this end, stock solutions were prepared at a concentration of 12.5 mg.mL^−1^ for each compound, from which volumes of 50, 100, and 150 µL were captured, added to tubes and, when necessary, completed with DMSO q.s.p. 150 µL (3%). Next, 100 µL (2%) of Tween 80 was added and completed with saline solution q.s.p. 5 mL. Then, ten healthy *A. salina* nauplii were placed inside each tube. After 24 h, the number of alive nauplii was counted. Concentration series (125, 250, and 375 µg·mL^−1^) of potassium dichromate solution was used as a positive control. Two negative controls were also prepared, one containing only saline solution, and another containing saline solution with 3% DMSO and 2% Tween 80. All experiments were performed in triplicate. Data are expressed in terms of mortality percentage versus concentration sample. Median lethal concentration (LC_50_) was estimated using a linear regression equation (Table S1).

### 2.6. Molecular Docking Study

Molecular docking studies were conducted using the Molegro Virtual Docker (MVD) 6.0 (Molexus IVS, Rørth Ellevej 3, Odder, Denmark) [31]. Three-dimensional (3D) structures of two macromolecules were retrieved from Protein Data Bank (PDB) “https://www.rcsb.org (15 March 2025)”: 50S ribosomal subunit (PDB ID: 1NJI) (active site coordinates: x = 50.3, y = 60.3, z = 70.1) [32] and sterol 14-alpha demethylase (CYP51) (PDB ID: 5TZ1) (active site coordinates: x = 70.65, y = 66.23, z = 4.36) [33]. These macromolecules were selected because they serve as targets for the control drugs used in both in vitro assays and docking studies.

The ligand structures were drawn using PerkinElmer ChemDraw Professional 16.0 (Springfield, MA, USA) and subsequently converted into 3D structures in the .sdf format using Open Babel 3.1.0 [34]. Root-mean-square deviation (RMSD) was calculated to assess the distance between the ligand and the protein-binding site during redocking. An RMSD value between 0–2 Å indicates a valid study [35].

Compound **3b**, Chloramphenicol and Fluconazole were subjected to molecular docking using MVD v. 6.0.1. All water molecules were removed from the enzyme structures, and both enzymes and compounds were prepared using predefined parameters. For the docking procedure (ligand–enzyme), a grid with a 15 Å radius and 0.30 Å resolution was applied, encompassing the binding site. A model was generated to evaluate the ligand–enzyme fit based on expected characteristics, using Moldock scoring (GRID) and search algorithms [31].

To visualize the interactions between compounds and active sites of the proteins, the Discovery Studio Visualizer 2017 17.2.0.16349 software was employed.

## 3. Results

### 3.1. Chemistry

Phthalimide and *N*-phthaloylglycine esters (**3a**–**d** and **4a**–**d**) were obtained by the synthetic route shown in Scheme 1.

Full data of precursors (**2a**–**d**) were found to be identical to the ones described previously [18,19]. Final products (**3a**–**d** and **4a**–**d**) were characterized by spectroscopic techniques of IR, ^1^H and ^13^C NMR, and HRMS.

### 3.2. In Silico/Vitro Antimicrobial Evaluation

Phthalimide and *N*-phthaloylglycine esters (**3a**–**d** and **4a**–**d**) had their in vitro antimicrobial activity evaluated against ten different strains, including Gram-(+) and Gram-(−) bacteria, yeast, and filamentous fungi, using the broth microdilution method to determine the MIC values, according to CLSI, documents M07-A10 [23] and M27-A3 [24]. Furthermore, in silico antimicrobial activity against *C. albicans* was acquired from the MolPredictX program [22]. In vitro assays and predicted data are available in Table 1 and Table 2.

### 3.3. Antifungal Mechanism of Action

Considering that compound **3b** showed significant antimicrobial activity against strains of *C. albicans*, the antifungal mechanism of action was investigated by sorbitol and ergosterol assays [25,26]. MIC values of compound **3b** in the absence and presence of sorbitol or ergosterol are available in Table 3.

### 3.4. Synergy Checkerboard Assay

Regarding antibacterial activity, compound **3b** showed the best results against strains of *S. aureus* and *P. aeruginosa.* Thus, the combined effect of compound **3b** with Chloramphenicol was studied, and FIC and FICI values are available in Table 4.

### 3.5. In Silico/Vitro Toxicity Evaluation

Considering compounds that showed significant antimicrobial activity, in silico/vitro toxicity evaluation was required for phthalimide and *N*-phthaloylglycine esters (**3b**, **3c**, **4a**, **4c**). The toxicity and lipophilicity prediction were acquired from the pkCSM program [29]. In vitro ecotoxicity on *Artemia salina* larvae was evaluated according to the adapted method reported by Sousa et al. [30], with some adjustments. A saline solution and a saline solution containing 3% DMSO and 2% Tween 80 were used as negative controls, in which any nauplius died, and a potassium dichromate solution was used as positive control, in which all nauplii died. In silico/vitro toxicity data are available in Table 5.

### 3.6. Molecular Docking Study

The results of the molecular docking study are summarized in the tables below. Table 6 provides detailed information on the crystalline structures of the enzymes, including PDB ID, macromolecule, species, PDB ligand, resolution, and RMSD values for the poses obtained through redocking. Table 7 presents the MolDock scores for the compounds docked into the active sites of the enzymes studied. These results offer valuable insights into the binding interactions and potential efficacy of the ligands with their respective targets.

## 4. Discussion

Two series of four compounds each were planned through two synthetic steps (Scheme 1). Firstly, according to a method previously reported [18,19], with some adjustments, precursor **2** was prepared by opening the THF ring with aroyl chloride **1** catalyzed by ZnCl_2_. Esters **2a**–**d** were obtained in yields ranging from 78 to 85% after purification by chromatographic column. In parallel, potassium salts of phthalimide (80% yield) and *N*-phthaloylglycine (96% yield) were prepared by basic alcoholysis of phthalimide and *N*-phthaloylglycine, respectively. Finally, final compounds **3** and **4** were obtained by the alkylation reaction between ester **2** and potassium salt of phthalimide or *N*-phthaloylglycine catalyzed by NaI. Phthalimide and *N*-phthaloylglycine esters (**3a**–**d** and **4a**–**d**) were obtained in yields ranging from 75 to 98% after purification by crystallization.

In NMR spectra of final compounds, the main signal that confirms the obtaining of products is that referring to the δ-position. In ^1^H NMR spectra, the triplet referring to Cl-CH_2_ appeared in the range of 3.58–3.62 ppm for precursors **2a**–**d**, while for final compounds **3a**–**d** and **4a**–**d**, the triplets referring to N-CH_2_ and O-CH_2_ appeared in the ranges of 3.77–3.84 and 4.24–4.25 ppm, respectively. This is due to the greater electronegativity of nitrogen/oxygen compared to chlorine, leading the shifts to a lower field. In IR spectra, the obtaining of final compounds was confirmed by the emergence of phthalimide carbonyl bands at 1770–1773 cm^−1^. Furthermore, an increase in symmetric/asymmetric stretching and in-plane/out-of-plane angular deformations relative to C–O/N at 1294–1016 cm^−1^ was observed.

With final compounds duly synthesized, purified, and characterized, an antimicrobial study was conducted. According to Table 1 and Table 2, with the exception of compound **3d** (R = Cl), which was not active against any strain tested, phthalimide esters (**3a**–**c**) showed activity against all ten different strains, including Gram-(+) and Gram-(−) bacteria, yeast, and filamentous fungi. On the other hand, with the exception of strains of filamentous fungi (*A. flavus* and *A. niger*), all *N*-phthaloylglycine esters (**4a**–**d**) showed activity against strains of Gram-(+) and Gram-(−) bacteria, and yeast fungi.

The best results were observed for compound **3b** (R = Me), especially against the Gram-(+) bacterium *S. aureus*, with MIC/MBC 128/512 µg·mL^−1^ (Chloramphenicol MIC/MBC 32/128 µg·mL^−1^), Gram-(−) bacterium *P. aeruginosa*, with MIC/MBC 128/512 µg·mL^−1^ (Chloramphenicol MIC/MBC 64/128 µg·mL^−1^), and yeast fungi *C. tropicalis*, with MIC/MFC 128/512 µg·mL^−1^ (Fluconazole MIC/MFC 256/512 µg·mL^−1^), and *C. albicans*, with MIC/MFC 128/512 µg·mL^−1^ (Fluconazole MIC/MFC 128/512 µg·mL^−1^). Furthermore, compared to *N*-phthaloylglycine pentyl ester previously reported by our research group [16], which presented MIC values equal to 256 and 1024 µg·mL^−1^ against strains of *C. tropicalis* ATCC 13803 and *E. coli* ATCC 25922, respectively, the antimicrobial activity seems to have been improved by adding butyl and aryl groups to phthalimide in the present work.

For *C. albicans*, the activity was evaluated from prediction to experiment. According to Table 2, there was a correlation between prediction and experiment only for compounds **3b**, **3c,** and **4d**. In silico studies express a trend, a probability, but the experiments are irreplaceable. Considering that compound **3b** was as or more active than Fluconazole against strains of *C. albicans*, the antifungal mechanism of action was investigated by sorbitol and ergosterol assays [25,26]. According to Table 3, the MIC values of compound **3b** in the presence of sorbitol did not change, while in the presence of ergosterol, the MIC values changed from 128 to 1024 µg·mL^−1^. In order to investigate a possible effect of compound **3b** on the integrity of the fungal cell wall, the culture medium was supplemented with sorbitol, an osmotic stabilizer for fungal protoplasts. The action of compound **3b** on the fungal cell membrane was also evaluated using an assay with ergosterol, a lipid naturally present in the fungal cell membrane. Thus, it seems that the antifungal mechanism of action occurs via ergosterol, as well as Fluconazole, which inhibits ergosterol biosynthesis in the fungal cell membrane [8].

Regarding the best results for antibacterial activity, the combined effect of compound **3b** with Chloramphenicol was studied against strains of *S. aureus* and *P. aeruginosa*. According to Table 4, a FICI value equal to 0.5 was found for *P. aeruginosa*, i.e., synergy, while for *S. aureus* was found a FICI value equal to 1.25, i.e., indifference. Therefore, the combination of compound **3b** with Chloramphenicol eliminated strains of *P. aeruginosa* at a lower concentration, emerging as a possible option for the treatment of infections caused by multidrug-resistant (MDR) strains of *P. aeruginosa*. The reduced concentration of Chloramphenicol when combined with compound **3b** may mitigate the common side effects of Chloramphenicol [39], which needs to be verified by further research, including clinical strain assays, cytotoxicity evaluation, in vivo assays, and clinical trials.

Finally, the four most promising compounds (with an MIC ≤ 512 µg·mL^−1^ against any strain) were selected to have their in silico/vitro toxicity evaluated. Thus, according to Table 5, with the exception of compound **4c** (R = OMe) and Fluconazole, compounds **3b**, **3c,** and **4a** and Chloramphenicol were predicted as mutagenic potential by the Ames test. With the exception of Chloramphenicol, all selected compounds (**3b**, **3c**, **4a**, **4c**), including Fluconazole, are potentially hepatotoxic. On the other hand, they were predicted not to cause skin sensitization, suggesting a potential application for topical use.

The minnow test predicts the median lethal concentration (LC_50_) for flathead minnows, and log LC_50_ values below −0.3 are predicted as high acute toxicity [29]. Then, with the exception of compound **4a** (R = H), compounds **3b**, **3c,** and **4c** are potentially toxic on flathead minnows. With regard to the results on *A. salina* larvae, compounds **3b** and **3c** are moderately toxic, with LC_50_ values between 100 and 500 µg·mL^−1^, while compounds **4a** and **4c** are non-toxic, with LC_50_ values ≥ 1000 µg·mL^−1^. Thus, there was a trend correlation between prediction and experiment for compounds **3b**, **3c,** and **4a**. Regarding ecotoxicity on *A. salina* larvae, there is no linear correlation, but it seems that more lipophilic compounds (log P ~ 3) tend to be more toxic. On the other hand, according to LC_50_ values from the literature (Table 5), Chloramphenicol and Fluconazole showed slight toxicity on *A. salina* larvae. Therefore, compounds **4a** and **4b** are less toxic on *A. salina* larvae than Chloramphenicol and Fluconazole.

In an attempt to understand the mechanism of action at the molecular level, a docking study was conducted with the most promising compound, **3b**, against the targets 50S ribosomal subunit (1NJI) and Sterol 14-alpha demethylase (CYP51) (5TZ1), which are molecular targets for the drugs used as controls in both in vitro tests and this in silico study.

**Figure 2:**
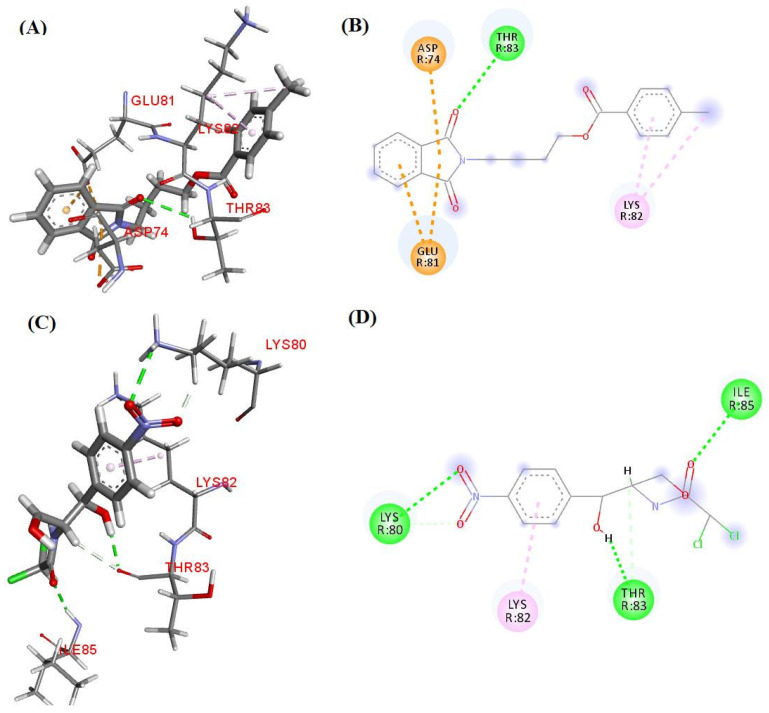
(**A**) The 3D structure of compound **3b**; (**B**) 2D structure of compound **3b**; (**C**) 3D structure of Chloramphenicol; (**D**) 3D structure of Chloramphenicol. Interactions performed by compounds **3b** and Chloramphenicol with 50S ribosomal subunit (1NJI) and the corresponding amino acid residues. Colors: Green (hydrogen bond), blue and pink (hydrophobic interaction), orange (π–Sulfur interaction). Residues: ASP (Aspartate), GLU (Glutamate), ILE (Isoleucine), LYS (Lysine), THR (Threonine).

The results obtained from the molecular docking study between compound **3b** and the target 1NJI, compared to Chloramphenicol, suggest that compound **3b** has a more favorable antibacterial mechanism of action from a thermodynamic perspective. The interaction energy of compound **3b** (−92.69 kcal·mol^−1^) was significantly more negative than that of Chloramphenicol (−73.13 kcal·mol^−1^), indicating greater stability in binding to the active site of the target protein (Table 7). This energy difference may be related to the interaction profile established by compound **3b**, which includes π–anion interactions with residues ASP R:74 and GLU R:81, as well as hydrogen bonds with THR R:83, and alkyl interactions with LYS R:82 (Figure 2).

The π–anion interactions observed for compound **3b** are particularly relevant, as they involve negatively charged residues (ASP R:74 and GLU R:81), which may contribute to greater specificity and affinity for the target protein. Additionally, the presence of alkyl interactions with LYS R:82 may further stabilize the protein–ligand complex. In contrast, Chloramphenicol exhibited hydrogen bonds with LYS R:80, THR R:83, and ILE R:85, as well as a π–alkyl interaction with LYS R:82, which, although relevant, appears to be less effective in terms of binding energy (Figure 2).

These results suggest that compound **3b** may act as a promising candidate for the development of novel antibacterial agents, surpassing Chloramphenicol in terms of binding affinity and stability. The elucidation of the specific molecular interactions of compound **3b** with the target 1NJI paves the way for future structural optimization and experimental validation studies, aiming at the discovery of more effective drugs against resistant pathogens.

The results of the molecular docking study between compound **3b** and the target 5TZ1, compared to Fluconazole, reveal that compound **3b** exhibits slightly less favorable interaction energy (−107.25 kcal·mol^−1^) than that of Fluconazole (−113.86 kcal·mol^−1^). However, the interaction profile established by compound **3b** suggests a promising and potentially effective binding to the active site of the target protein, indicating a distinct and relevant antifungal mechanism of action (Table 7).

**Figure 3:**
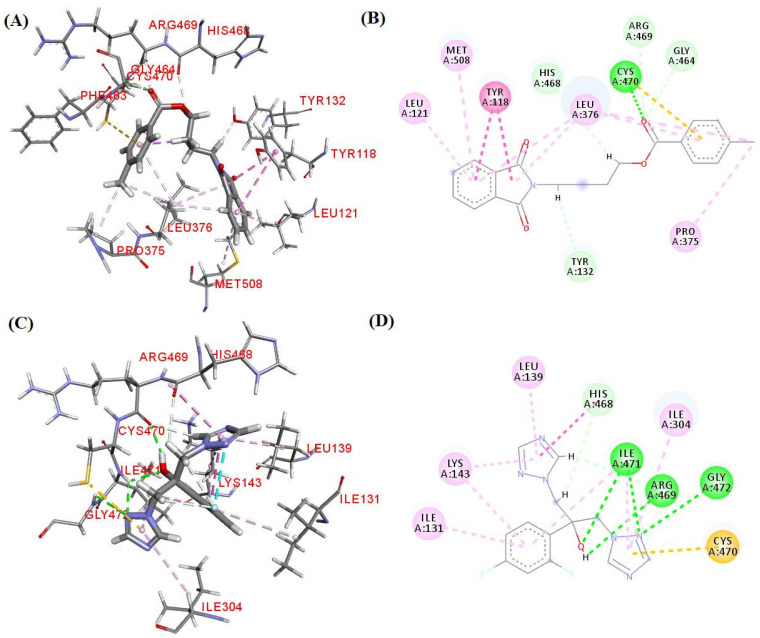
(**A**) The 3D structure of compound **3b**; (**B**) 2D structure of compound **3b**; (**C**) 3D structure of Fluconazole; (**D**) 2D structure of Fluconazole. Interactions performed by compounds **3b** and Fluconazole with Sterol 14-alpha demethylase (CYP51) (5TZ1) and the corresponding amino acid residues. Colors: Green (hydrogen bond), blue and pink (hydrophobic interaction), orange (π–Sulfur interaction). Residues: ARG (Arginine), CYS (Cysteine), GLY (Glycine), HIS (Histidine), ILE (Isoleucine), LEU (Leucine), LYS (Lysine), MET (Methionine), PRO (Proline), TYR (Tyrosine).

Fluconazole demonstrated hydrogen bonds with ILE A:471, ARG A:469, and GLY A:472, as well as a π–sulfur interaction with CYS A:470, and hydrophobic interactions with HIS A:468, ILE A:131, LYS A:143, LEU A:139, and ILE A:304. These interactions are characteristic of its binding to the target enzyme, contributing to its antifungal efficacy. On the other hand, compound **3b** established hydrogen bonds and a π–sulfur interaction with CYS A:470, standing out for the formation of a π–sulfur bond, which is less common and may confer greater specificity to the protein–ligand complex. Additionally, compound **3b** exhibited a broad profile of hydrophobic interactions with residues such as HIS A:468, ARG A:469, GLY A:464, TYR A:132, TYR A:118, LEU A:121, MET A:508, LEU A:376, and PRO A:375, suggesting possible additional stabilization of the complex (Figure 3).

Although the interaction energy of compound **3b** is slightly less favorable than that of Fluconazole, the diversity and nature of the observed interactions suggest that compound **3b** may act through a complementary or alternative mechanism, potentially useful against Fluconazole-resistant strains. The π–sulfur interaction with CYS A:470, in particular, is a notable highlight, as it may contribute to a more specific and stable binding (Figure 3). These results indicate that compound **3b** is a viable candidate for future optimization and antifungal evaluation studies, potentially representing a new therapeutic strategy in the fight against fungal infections.

## 5. Conclusions

In conclusion, two series of phthalimide and *N*-phthaloylglycine esters were designed to investigate whether the addition of butyl and aryl groups enhances their antimicrobial properties. The best results were observed for phthalimide aryl ester **3b** (R = Me), which showed significant antimicrobial activity against Gram-(+) and Gram-(−) bacteria, *S. aureus* and *P. aeruginosa*, respectively, and yeast fungi, *C. tropicalis* and *C. albicans*, with MIC values equal to 128 µg·mL^−1^. Considering that compound **3b** was as or more active than Fluconazole against strains of *C. albicans*, its antifungal mechanism of action was studied, where MIC values changed from 128 to 1024 µg·mL^−1^ in the presence of ergosterol. Furthermore, compound **3b** showed synergy with Chloramphenicol against *P. aeruginosa*, with a FICI value equal to 0.5. Finally, considering the potential disposal of drugs in the aquatic environment, the four most promising compounds had their ecotoxicity evaluated, which showed moderate toxicity (**3b** and **3c**) to non-toxicity (**4a** and **4c**) on *A. salina* larvae. The molecular docking results demonstrated that compound **3b** exhibits enhanced binding affinity and stability with the 50S ribosomal subunit (−92.69 kcal·mol^−1^) compared to Chloramphenicol, along with a distinctive π–sulfur interaction with CYP51, highlighting its potential as a promising dual-action antibacterial and antifungal agent against resistant pathogens.

## Figures and Tables

**Table 1:** In vitro antimicrobial evaluation of phthalimide and *N*-phthaloylglycine esters (**3a**–**d** and **4a**–**d**).

Compound	R	Bacteria MIC/MBC *				Fungi MIC/MFC *			
		Gram-(+)		Gram-(−)		Yeast		Filamentous	
		*S. aureus*		*E. coli*	*P. aeruginosa*	*C. tropicalis*		*A. flavus*	*A. niger*
		ATCC 25923	LM 01	ATCC 25922	ATCC 25853	ATCC 13803	LM 303	ATCC 4603	ATCC 6275
**3a**	H	1024/−	1024/−	1024/−	1024/−	1024/−	1024/−	1024/−	1024/−
**3b**	Me	128/512	128/512	256/512	128/512	128/512	256/512	1024/−	1024/−
**3c**	OMe	512/−	512/−	512/−	512/−	256/1024	256/1024	1024/1024	1024/1024
**3d**	Cl	−/−	−/−	−/−	−/−	−/−	−/−	−/−	−/−
**4a**	H	256/1024	256/1024	256/1024	512/1024	256/512	256/512	−/−	−/−
**4b**	Me	1024/−	1024/−	1024/−	1024/−	1024/−	1024/−	−/−	−/−
**4c**	OMe	512/−	512/−	512/−	1024/−	512/−	512/−	−/−	−/−
**4d**	Cl	1024/−	1024/−	1024/−	1024/−	1024/−	1024/−	−/−	−/−
Chloramphenicol	NA	32/128	32/128	64/128	64/128	NA	NA	NA	NA
Fluconazole	NA	NA	NA	NA	NA	256/512	256/512	256/1024	512/1024
Culture medium	NA	−/−	−/−	−/−	−/−	−/−	−/−	−/−	−/−

MIC: Minimum Inhibitory Concentration; MBC: Minimum Bactericidal Concentration; MFC: Minimum Fungicidal Concentration; NA: Not Applicable; (−): Not active in the concentration range of 1024–0.5 μg.mL^−1^. * µg·mL^−1^. All experiments were performed in triplicate.

**Table 2:** In silico/vitro antimicrobial evaluation of phthalimide and *N*-phthaloylglycine esters (**3a**–**d** and **4a**–**d**).

Compound	R	Yeast Fungus *C. albicans*			
		In Silico		In Vitro MIC/MFC *	
		Predicted Outcome	Probability Active (%)	ATCC 76485	LM 65
**3a**	H	Inactive	40	1024/−	1024/−
**3b**	Me	Active	60	128/512	128/512
**3c**	OMe	Active	100	256/1024	256/1024
**3d**	Cl	Active	100	−/−	−/−
**4a**	H	Inactive	20	256/512	256/512
**4b**	Me	Inactive	20	1024/−	1024/−
**4c**	OMe	Inactive	20	512/−	512/−
**4d**	Cl	Active	60	1024/−	1024/−
Fluconazole	NA	Active	100	128/512	256/512
Culture medium	NA	NA	NA	−/−	−/−

MIC: Minimum Inhibitory Concentration; MFC: Minimum Fungicidal Concentration; NA: Not Applicable; (−): Not active in the concentration range of 1024–0.5 μg.mL^−1^. * µg·mL^−1^. In vitro assays were performed in triplicate.

**Table 3:** MIC * values of compound **3b** in the absence and presence of sorbitol ** or ergosterol ***.

Compound	*C. albicans* ATCC 76485			
	Sorbitol		Ergosterol	
	Absence	Presence	Absence	Presence
**3b**	128	128	128	1024
Caspofungin	0.5	2	NA	NA
Fluconazole	NA	NA	128	512
Culture medium	−/−	−/−	−/−	−/−

MIC: Minimum Inhibitory Concentration; NA: Not Applicable; (−): Not active in the concentration range of 1024–0.5 μg.mL^−1^. * µg·mL^−1^. ** 0.8 M. *** 400 µg·mL^−1^. All experiments were performed in duplicate.

**Table 4:** FIC * and FICI values of compound **3b**.

Strains	FIC_A_	FIC_B_	FICI	Outcome
*S. aureus* **ATCC 25923**	0.25	1.0	1.25	Indifference
*P. aeruginosa* **ATCC 25853**	0.25	0.25	0.5	Synergy

FIC: Fractional Inhibitory Concentration; FIC_A_: FIC of compound **3b**; FIC_B_: FIC of Chloramphenicol; FICI: Fractional Inhibitory Concentration Index. * µg·mL^−1^. All experiments were performed in duplicate. Reference for FICI: synergy ≤ 0.5 < addition ≤ 1 < indifference ≤ 4 < antagonism [27,28].

**Table 5:** In silico/vitro toxicity evaluation of phthalimide and *N*-phthaloylglycine esters (**3b**, **3c**, **4a**, **4c**).

Compound	R	Toxicity					Lipophilicity
		In Silico				In Vitro	In Silico
		Ames	Hepato	Skin Sensitization	Minnow Log LC_50_	*Artemia salina* LC_50_ *	Log P
**3b**	Me	Yes	Yes	No	−1.895	239.000	3.228
**3c**	OMe	Yes	Yes	No	−1.784	224.095	2.928
**4a**	H	Yes	Yes	No	0.455	1028.33	2.463
**4c**	OMe	No	Yes	No	−0.497	>>1000	2.471
Chloramphenicol	NA	Yes	No	No	1.892	>20 [36]	0.909
Fluconazole	NA	No	Yes	No	3.872	802.28 [37]	0.735

LC_50_: Median Lethal Concentration; Log P: octanol/water partition coefficient; NA: Not Applicable. * µg·mL^−1^. Note: categorical (Yes/No) and numeric (log LC_50_) unit. Reference for LC_50_: highly toxic < 100 ≤ moderately toxic < 500 ≤ slightly toxic < 1000 ≤ non-toxic [38].

**Table 6:** Information on the crystalline structures of enzymes and root-mean-square deviation (RMSD) values for the poses obtained by redocking.

PDB ID	Macromolecule	Species	PDB Ligand	Resolution	RMSD
1NJI	50S ribosomal subunit	*Haloarcula marismortui*	CLM_9001	3.00 Å	2.21 Å
5TZ1	Sterol 14-alpha demethylase (CYP51)	*Candida albicans*	VT1_602	2.00 Å	1.87 Å

**Table 7:** MolDock scores (kcal·mol^−1^) for enzymes studied.

Compound	50S Ribosomal Subunit	Sterol 14-Alpha Demethylase (CYP51)
**3b**	−92.69	−107.25
Chloramphenicol	−73.13	−
Fluconazole	−	−113.86
CLM_9001	−73.13	−
VT1_602	−	−112.54

## Data Availability

The original contributions presented in this study are included in the article/Supplementary Materials. Further inquiries can be directed to the corresponding author.

## Supplementary Materials

The following supporting information can be downloaded at: https://www.mdpi.com/article/10.3390/life15040518/s1, Figure S1. ^1^H NMR spectrum (400 MHz, CDCl_3_) of compound **3a**. Figure S2. ^13^C Attached Proton Test (APT) spectrum (100 MHz, CDCl_3_) of compound **3a**. Figure S3. IR spectrum (KBr) of compound **3a**. Figure S4. HRMS spectrum (ESI) of compound **3a**. Figure S5. ^1^H NMR spectrum (500 MHz, CDCl_3_) of compound **3b**. Figure S6. ^13^C NMR spectrum (125 MHz, CDCl_3_) of compound **3b**. Figure S7. IR spectrum (KBr) of compound **3b**. Figure S8. HRMS spectrum (ESI) of compound **3b**. Figure S9. ^1^H NMR spectrum (500 MHz, CDCl_3_) of compound **3c**. Figure S10. ^13^C NMR spectrum (125 MHz, CDCl_3_) of compound **3c**. Figure S11. IR spectrum (KBr) of compound **3c**. Figure S12. HRMS spectrum (ESI) of compound **3c**. Figure S13. ^1^H NMR spectrum (400 MHz, CDCl_3_) of compound **3d**. Figure S14. ^13^C NMR spectrum (100 MHz, CDCl_3_) of compound **3d**. Figure S15**.** IR spectrum (KBr) of compound **3d**. Figure S16. HRMS spectrum (ESI) of compound **3d**. Figure S17. ^1^H NMR spectrum (500 MHz, CDCl_3_) of compound **4a**. Figure S18. ^13^C APT spectrum (125 MHz, CDCl_3_) of compound **4a**. Figure S19**.** IR spectrum (ATR) of compound **4a**. Figure S20. HRMS spectrum (ESI) of compound **4a**. Figure S21**.** ^1^H NMR spectrum (400 MHz, CDCl_3_) of compound **4b**. Figure S22. ^13^C APT spectrum (100 MHz, CDCl_3_) of compound **4b**. Figure S23. IR spectrum (KBr) of compound **4b**. Figure S24. HRMS spectrum (ESI) of compound **4b**. Figure S25. ^1^H NMR spectrum (400 MHz, CDCl_3_) of compound **4c**. Figure S26. ^13^C NMR spectrum (100 MHz, CDCl_3_) of compound **4c**. Figure S27. IR spectrum (KBr) of compound **4c**. Figure S28. HRMS spectrum (ESI) of compound **4c**. Figure S29. ^1^H NMR spectrum (400 MHz, CDCl_3_) of compound **4d**. Figure S30. ^13^C NMR spectrum (100 MHz, CDCl_3_) of compound **4d**. Figure S31**.** IR spectrum (ATR) of compound **4d**. Figure S32. HRMS spectrum (ESI) of compound **4d**. Table S1. Estimated median lethal concentration (LC_50_) for phthalimide and phthaloylglycine esters (**3b**, **3c**, **4a**, **4c**) on *Artemia salina* larvae using linear regression equation.
