# Supplementary material for: Antimicrobial Investigation of Phthalimide and N-Phthaloylglycine Esters: Activity, Mechanism of Action, Synergism and Ecotoxicity
Source: Life (Basel). 2025 Mar 21;15(4):518. doi: 10.3390/life15040518 (PMC12028801; doi:10.3390/life15040518)
Supplement: Supplementary file 1 [file life-15-00518-s001.zip › life-3525115-supplementary.pdf]

life

MDPI

Supplementary Material

Full NMR, IR and HRMS spectra for phthalimide and phthaloylglycine esters (**3a-d** and **4a-d**) are available in Figures S1–S32.

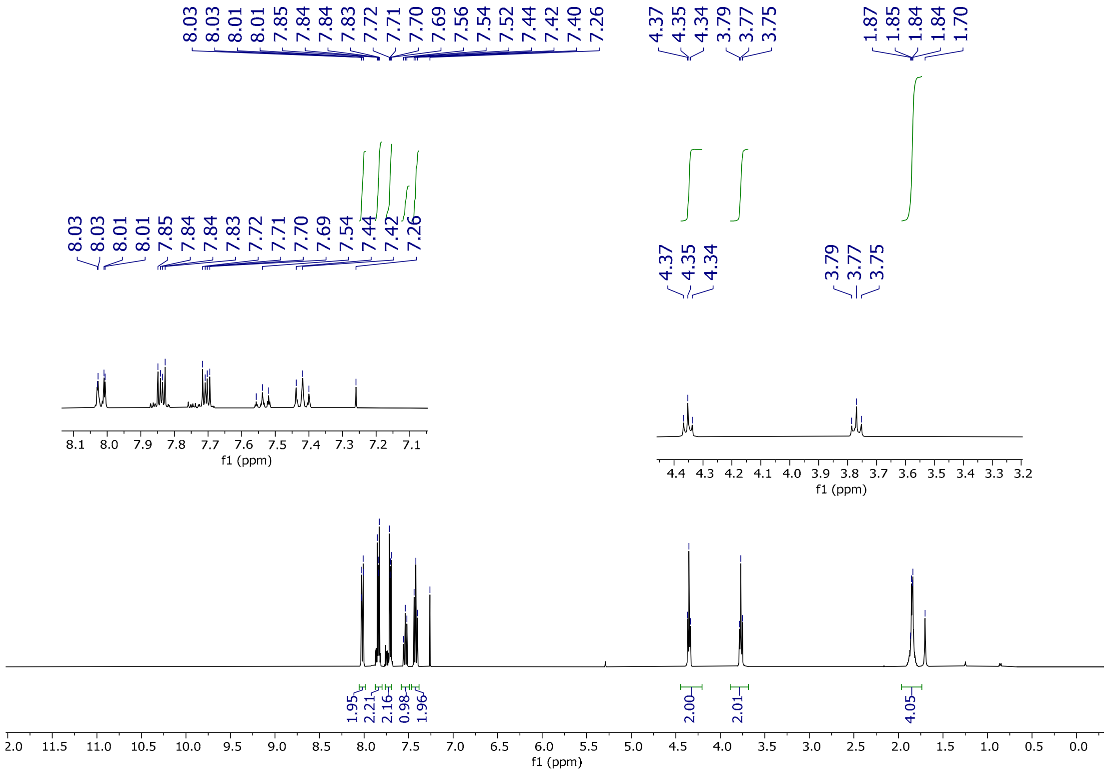

Figure S1. ^{1}H NMR spectrum (400 MHz, $CDCl_3$) of compound **3a**.

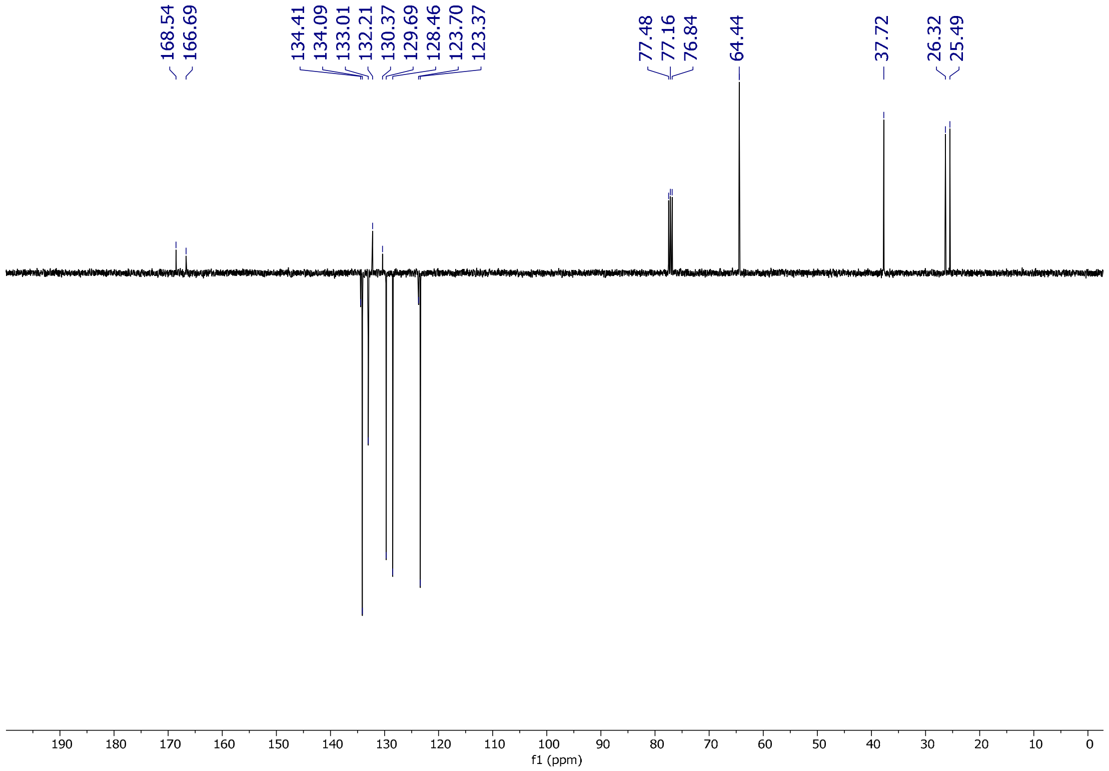

Figure S2. [13]C Attached Proton Test (APT) spectrum (100 MHz, $CDCl_3$) of compound **3a**.

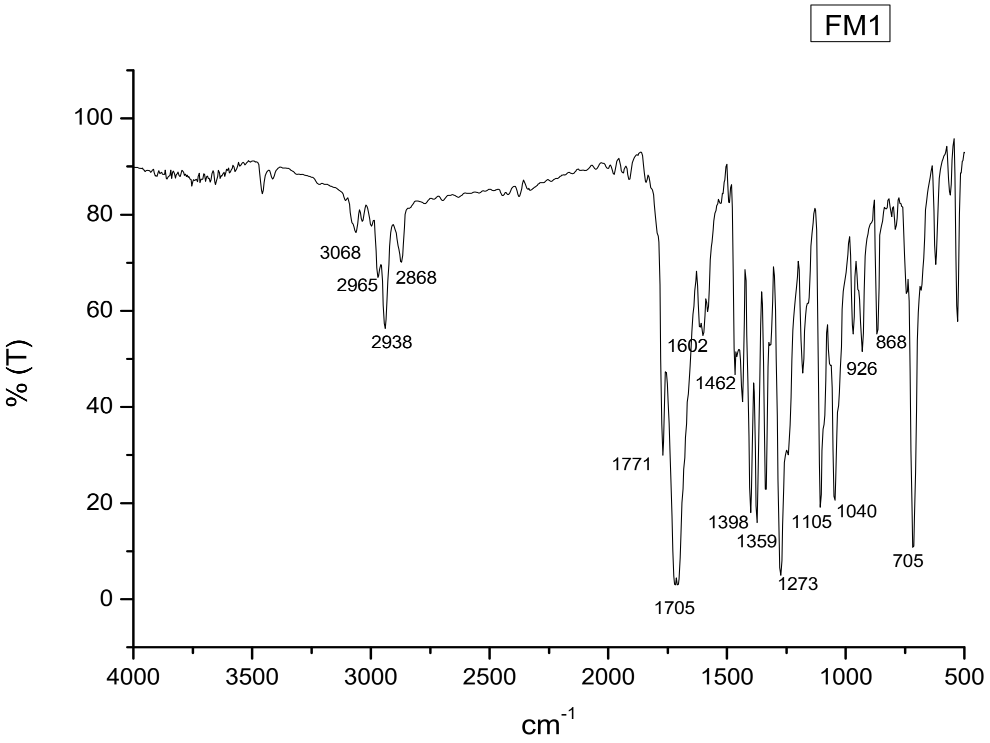

Figure S3. IR spectrum (KBr) of compound **3a**.

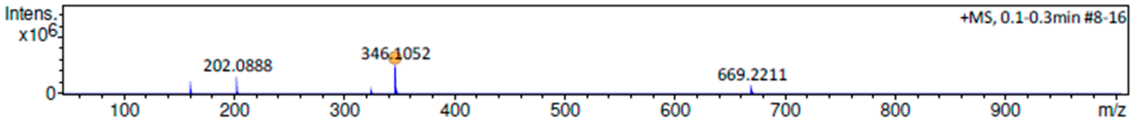

Meas. m/z	#	Ion Formula	m/z	err [ppm]	mSigma	# mSigma	Score	rdb	e^- Conf	N-Rule
346.1052	1	C19H17NNaO4	346.1050	-0.6	1.6	1	100.00	11.5	even	ok

Figure S4. HRMS spectrum (ESI) of compound **3a**.

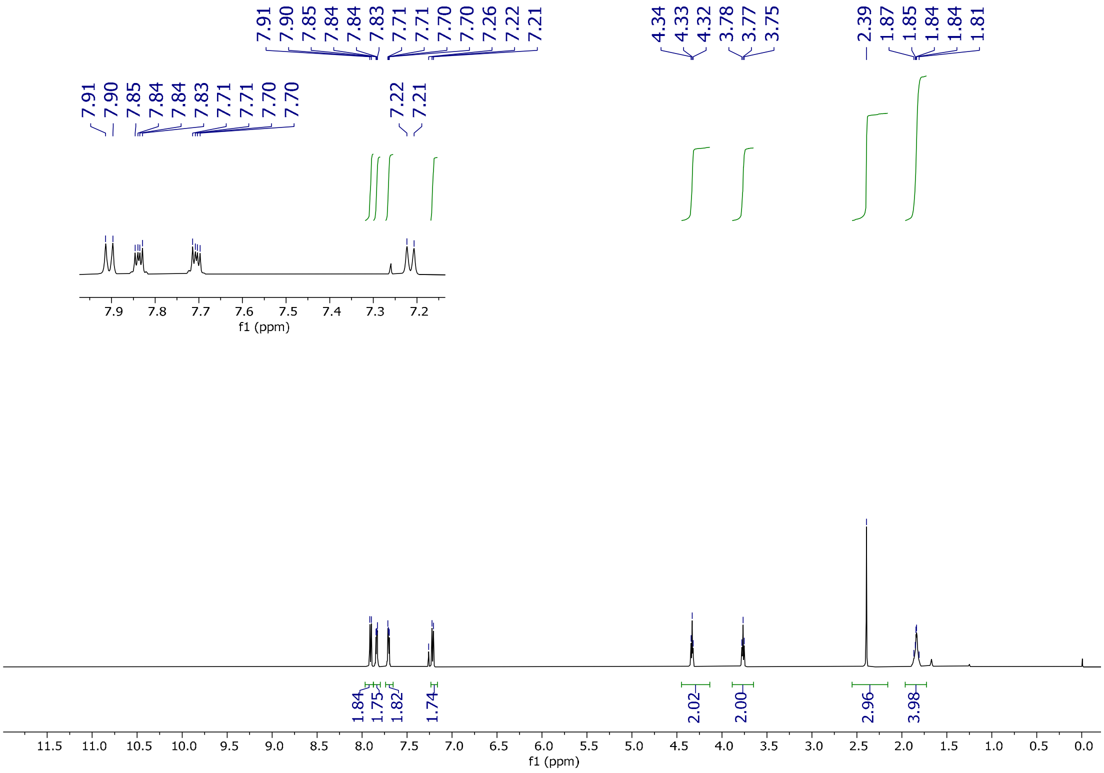

Figure S5. ^{1}H NMR spectrum (500 MHz, $CDCl_3$) of compound **3b**.

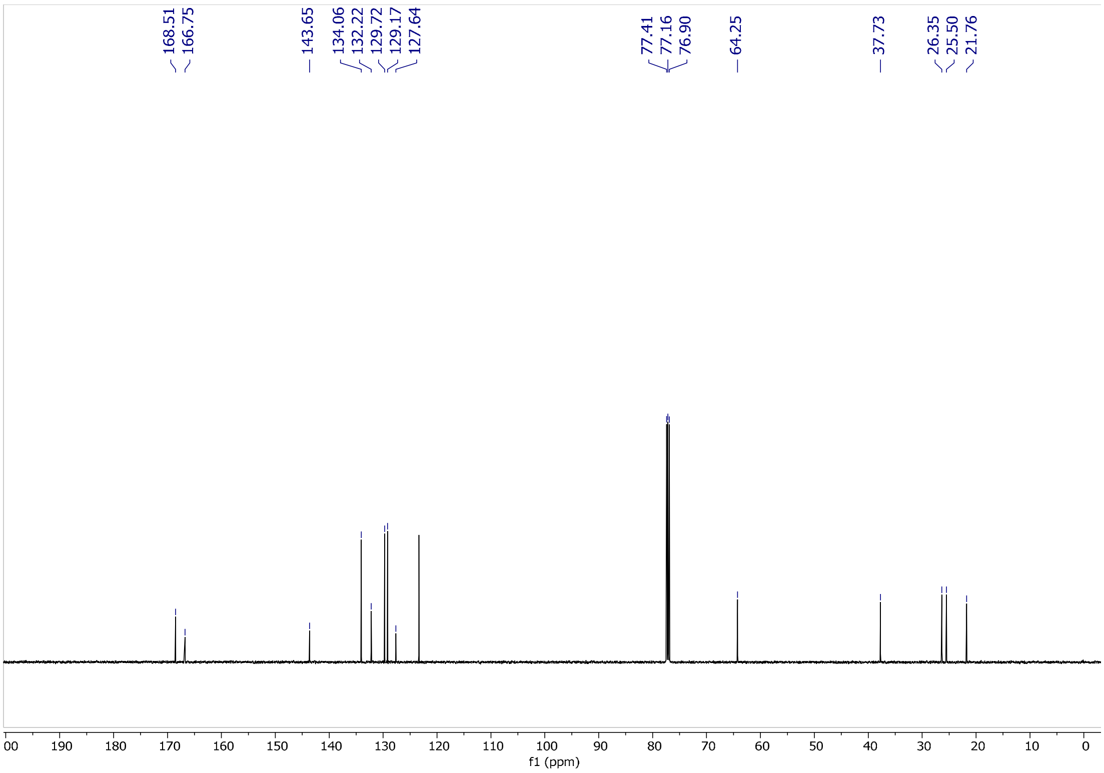

Figure S6. ^{13}C NMR spectrum (125 MHz, $CDCl_3$) of compound **3b**.

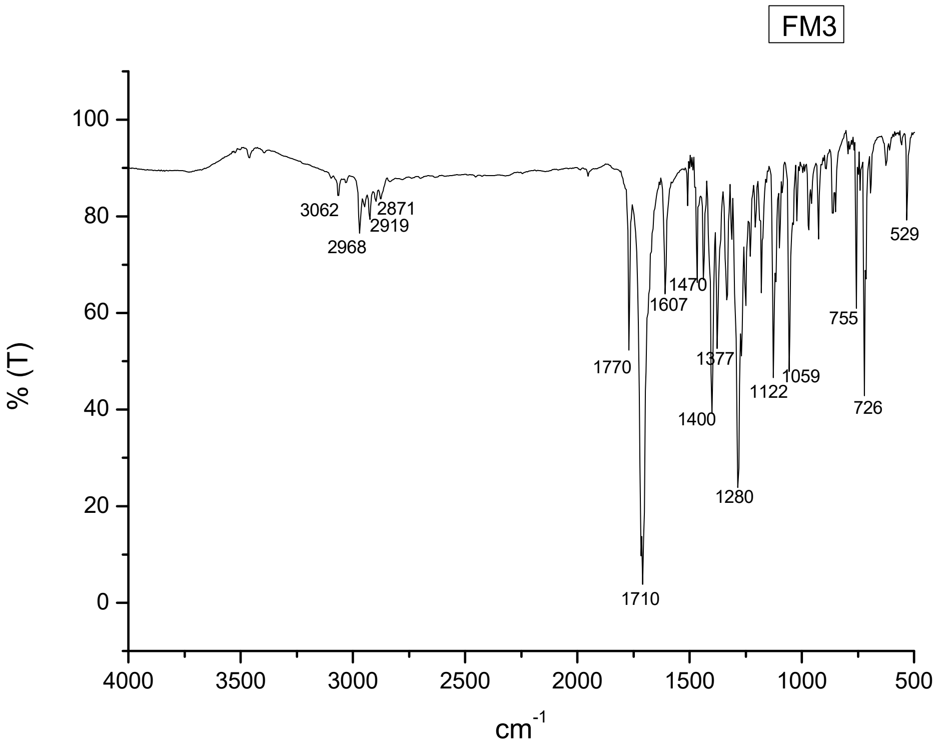

Figure S7. IR spectrum (KBr) of compound **3b**.

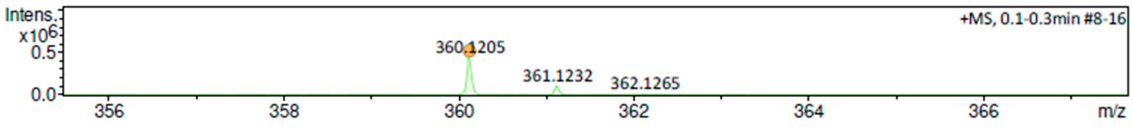

Meas. m/z	#	Ion Formula	m/z	err [ppm]	mSigma	# mSigma	Score	rdb	e⁻ Conf	N-Rule
360.1205	1	C20H19NNaO4	360.1206	0.4	3.3	1	100.00	11.5	even	ok

Figure S8. HRMS spectrum (ESI) of compound **3b**.

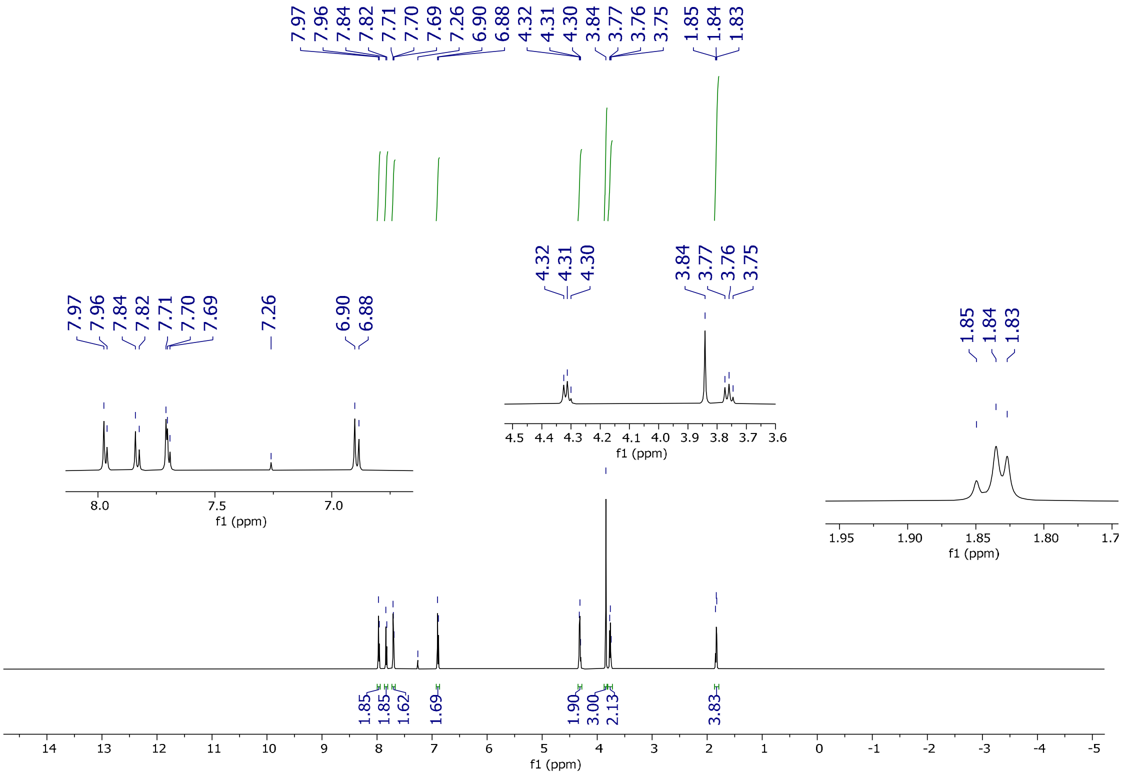

Figure S9. ^{1}H NMR spectrum (500 MHz, $CDCl_3$) of compound **3c**.

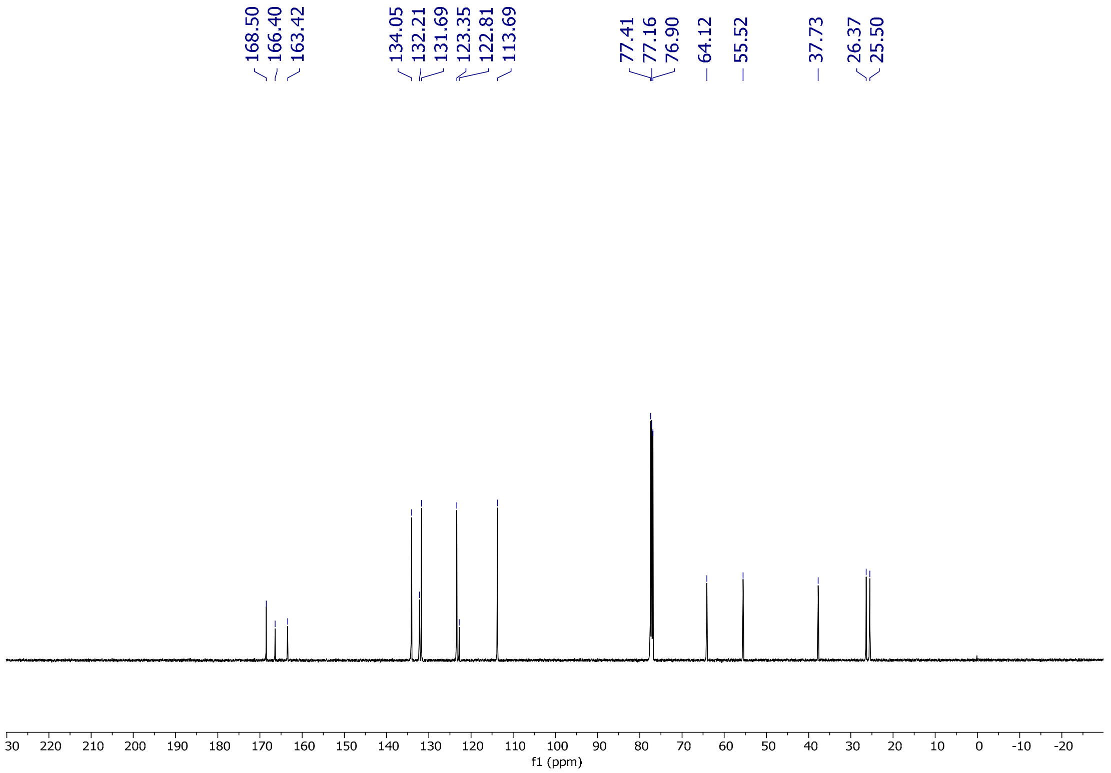

Figure S10. ^{13}C NMR spectrum (125 MHz, $CDCl_3$) of compound **3c**.

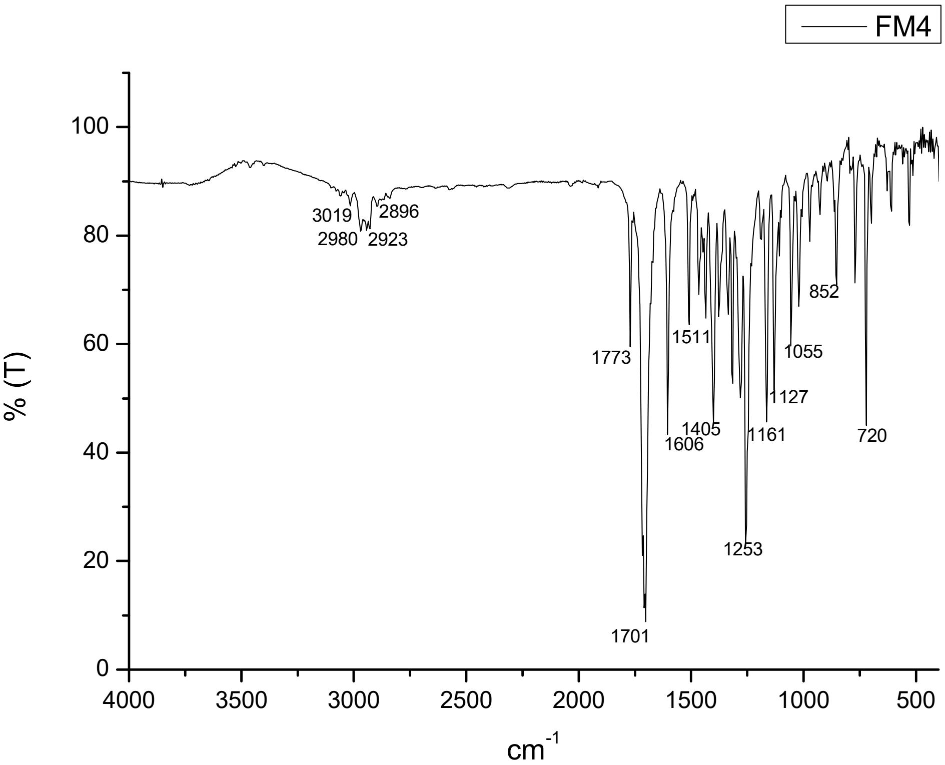

Figure S11. IR spectrum (KBr) of compound **3c**.

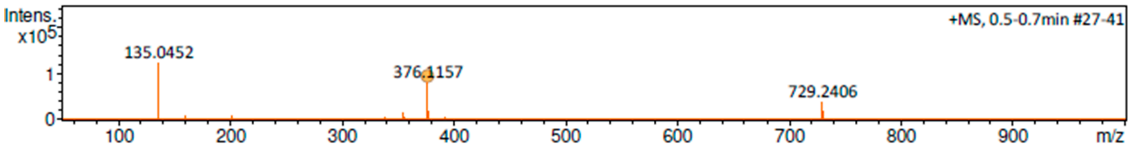

Meas. m/z	#	Ion Formula	m/z	err [ppm]	mSigma	# mSigma	Score	rdb	e⁻ Conf	N-Rule
376.1157	1	C20H19NNaO5	376.1155	-0.5	0.6	1	100.00	11.5	even	ok

Figure S12. HRMS spectrum (ESI) of compound **3c**.

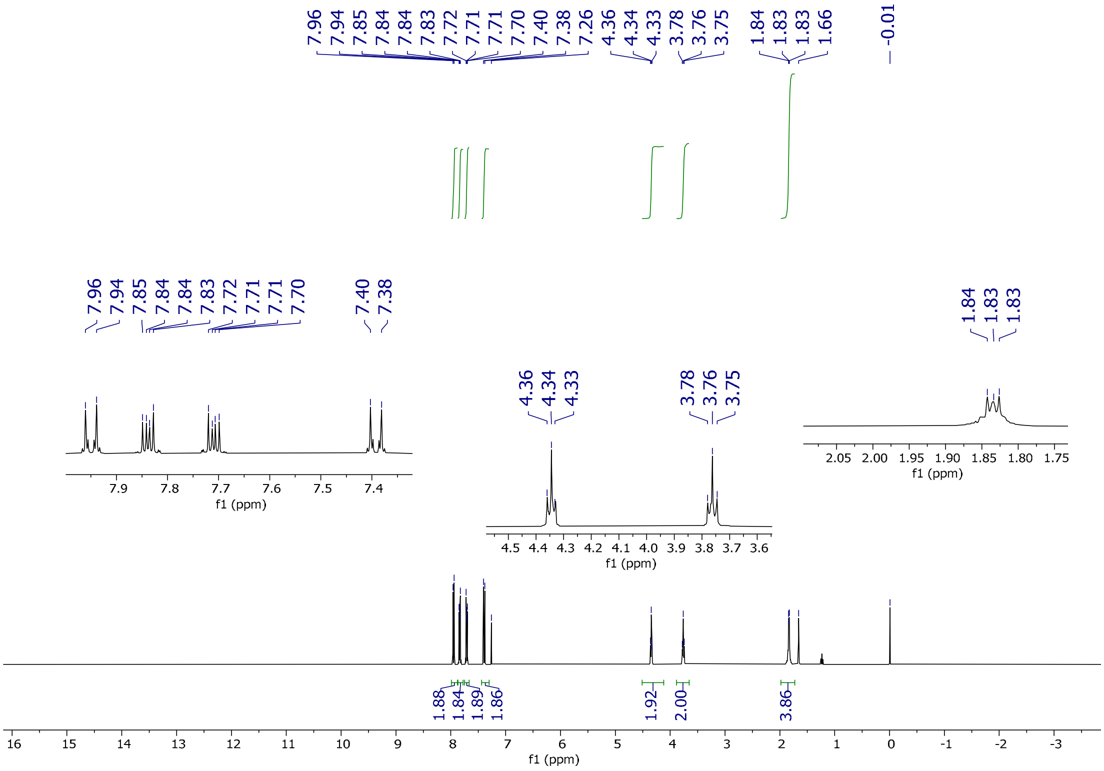

Figure S13. ^{1}H NMR spectrum (400 MHz, $CDCl_3$) of compound **3d**.

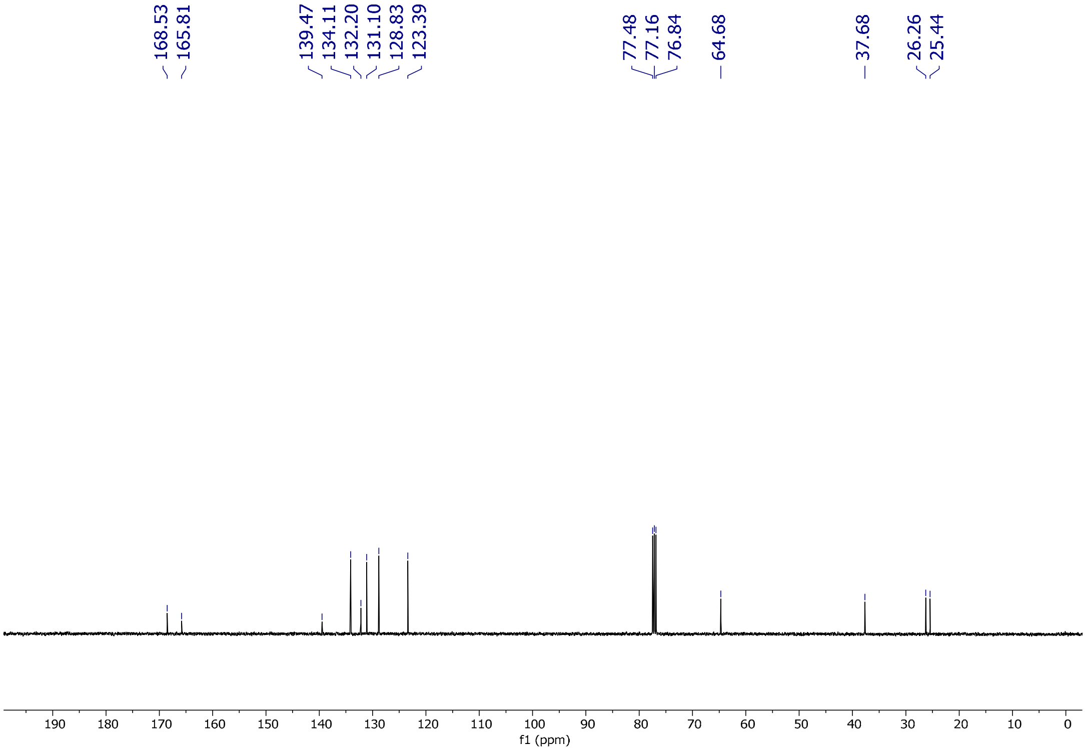

Figure S14. ^{13}C NMR spectrum (100 MHz, $CDCl_3$) of compound **3d**.

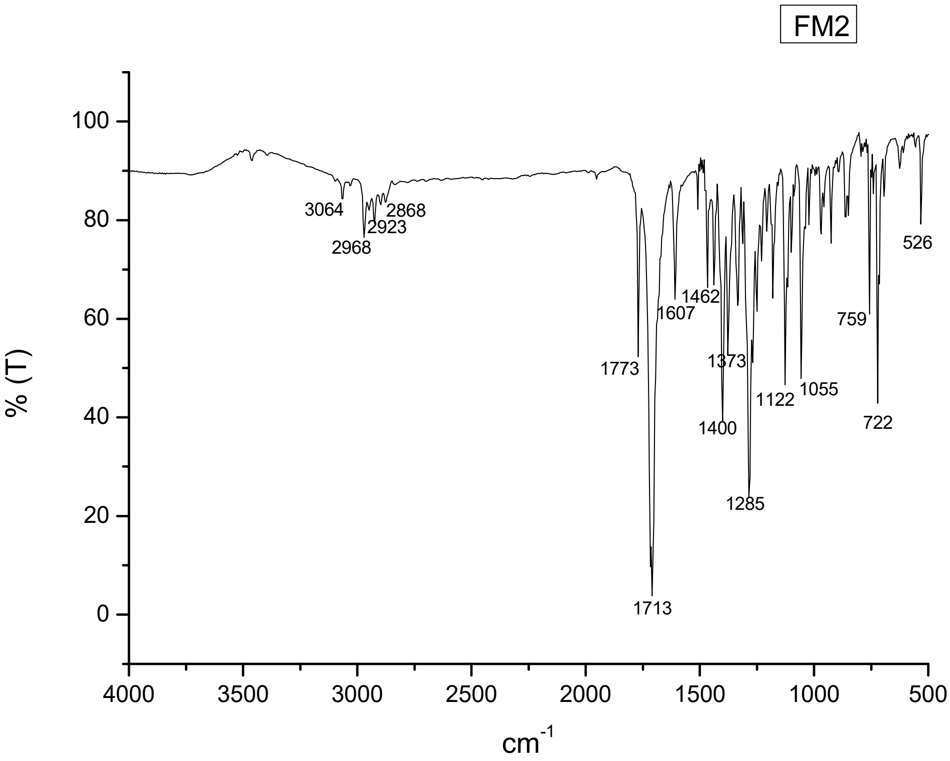

Figure S15. IR spectrum (KBr) of compound **3d**.

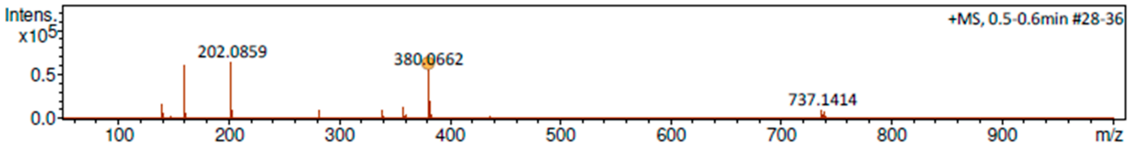

Meas. m/z	#	Ion Formula	m/z	err [ppm]	mSigma	# mSigma	Score	rdb	e^{-} Conf	N-Rule
380.0662	1	C19H16ClNNaO4	380.0660	-0.5	6.0	1	100.00	11.5	even	ok

Figure S16. HRMS spectrum (ESI) of compound **3d**.

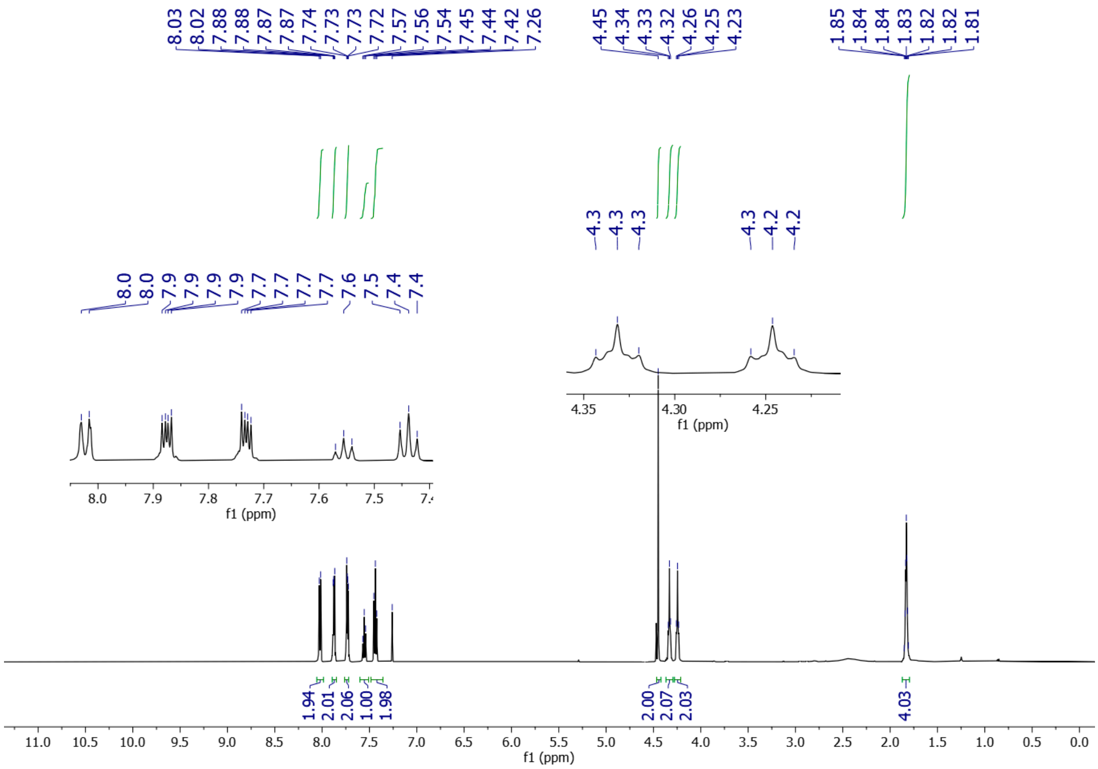

Figure S17. ^{1}H NMR spectrum (500 MHz, $CDCl_3$) of compound **4a**.

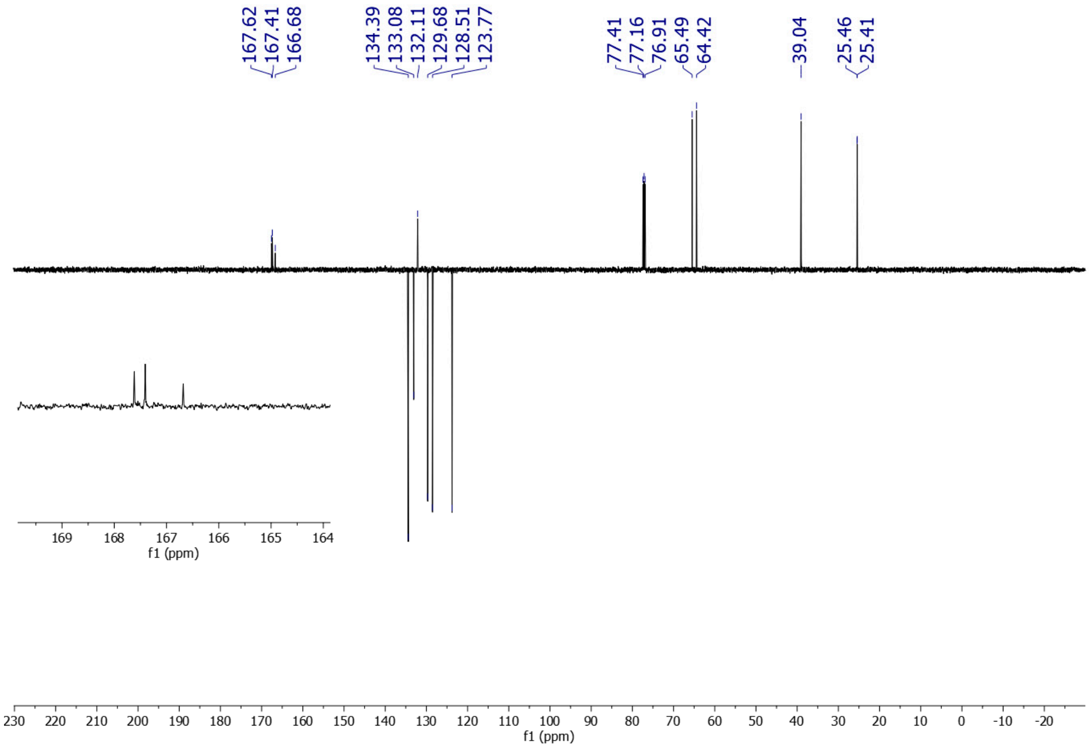

Figure S18. ^{13}C APT spectrum (125 MHz, $CDCl_3$) of compound **4a**.

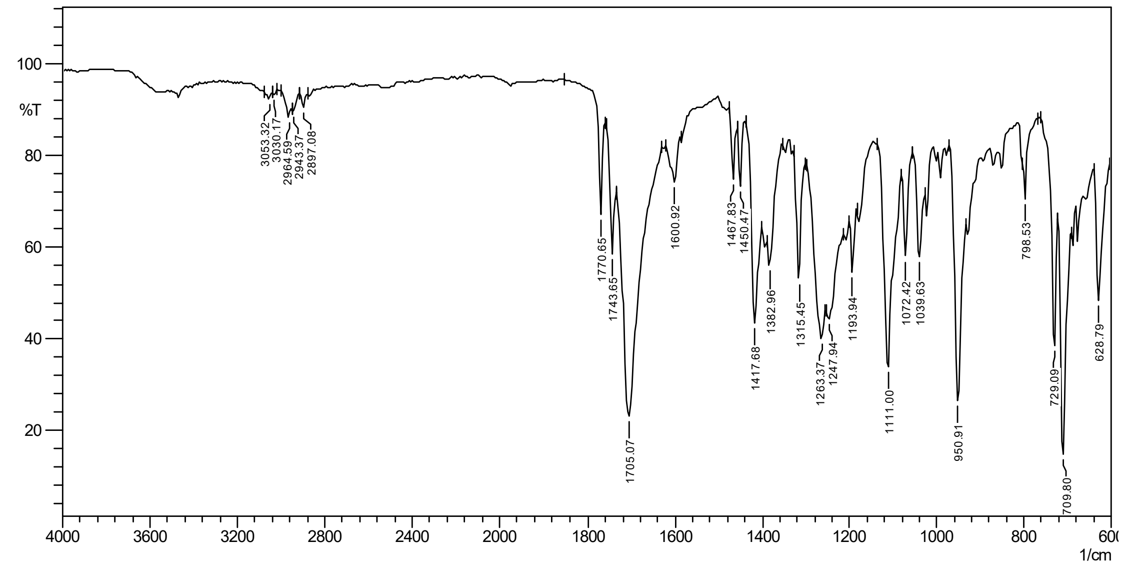

Figure S19. IR spectrum (ATR) of compound **4a**.

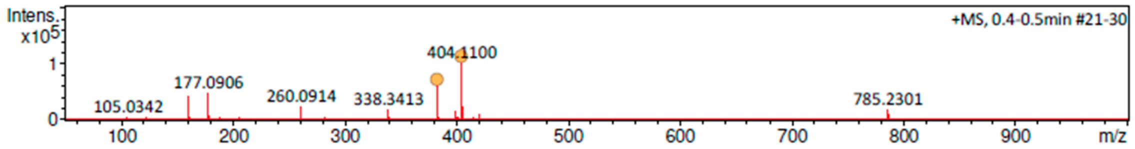

Meas. m/z	#	Ion Formula	m/z	err [ppm]	mSigma	# mSigma	Score	rdb	e⁻ Conf	N-Rule
382.1284	1	C21H20NO6	382.1285	0.2	6.8	1	100.00	12.5	even	ok
404.1100	1	C21H19NNaO6	404.1105	1.2	1.6	1	100.00	12.5	even	ok

Figure S20. HRMS spectrum (ESI) of compound **4a**.

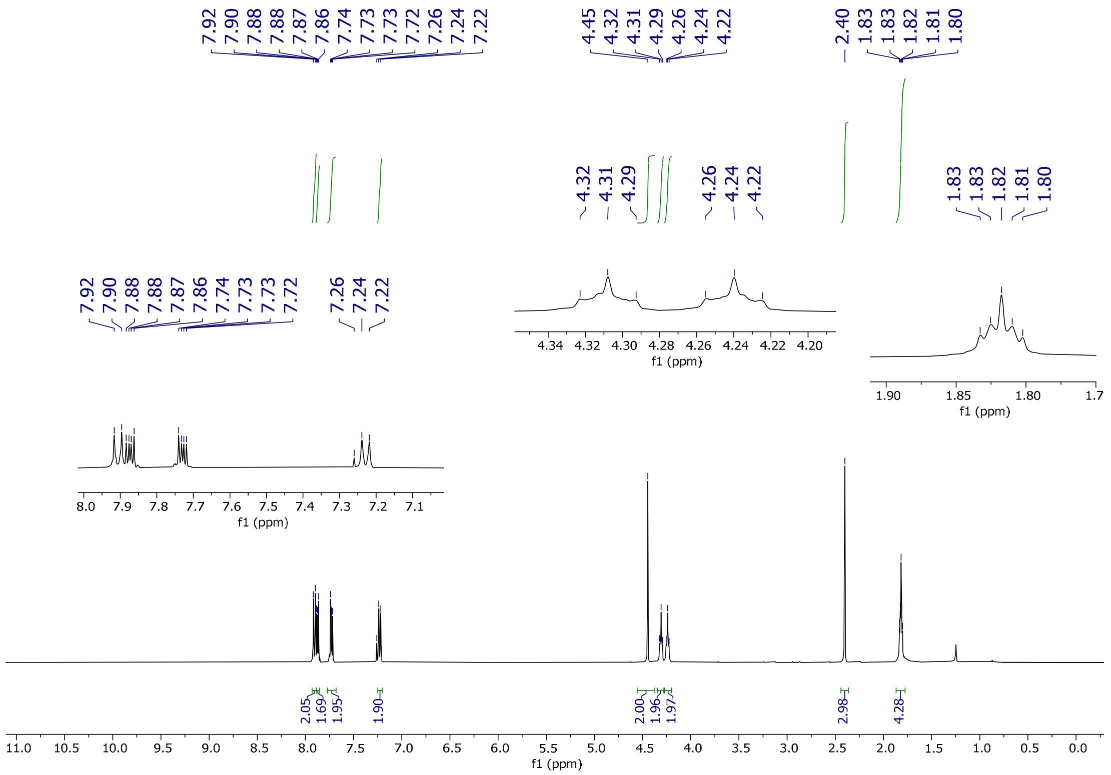

Figure S21. ¹H NMR spectrum (400 MHz, $CDCl_3$) of compound **4b**.

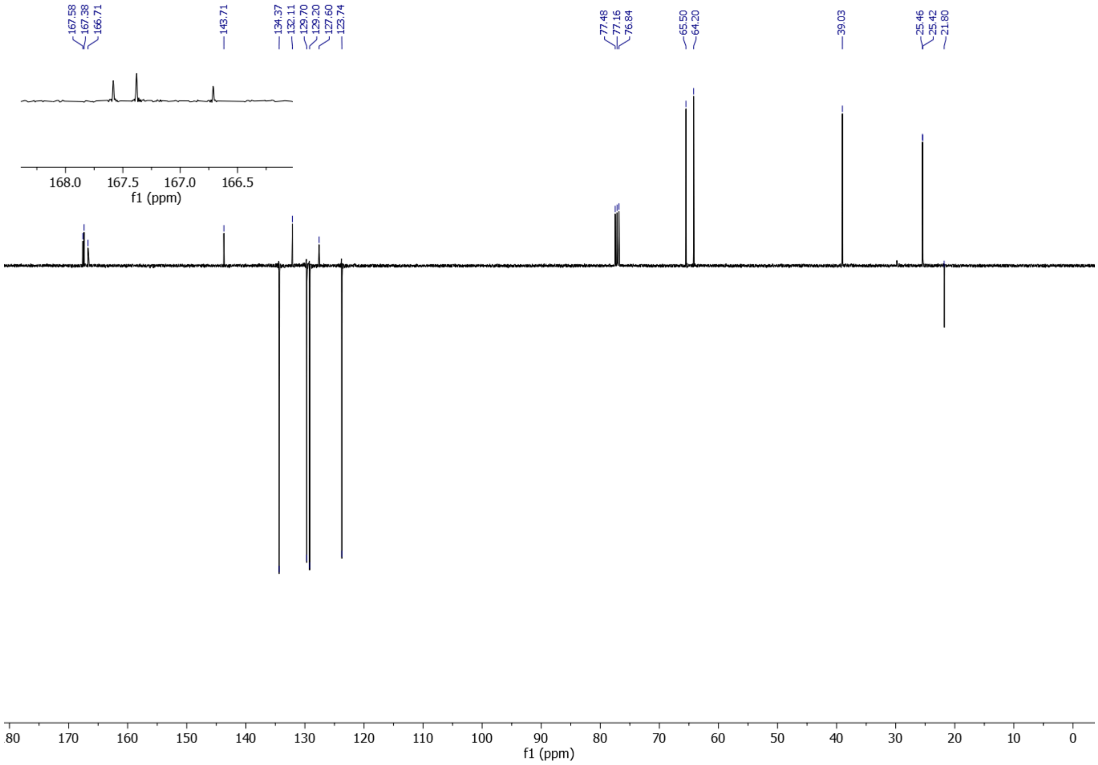

Figure S22. ^{13}C APT spectrum (100 MHz, $CDCl_3$) of compound **4b**.

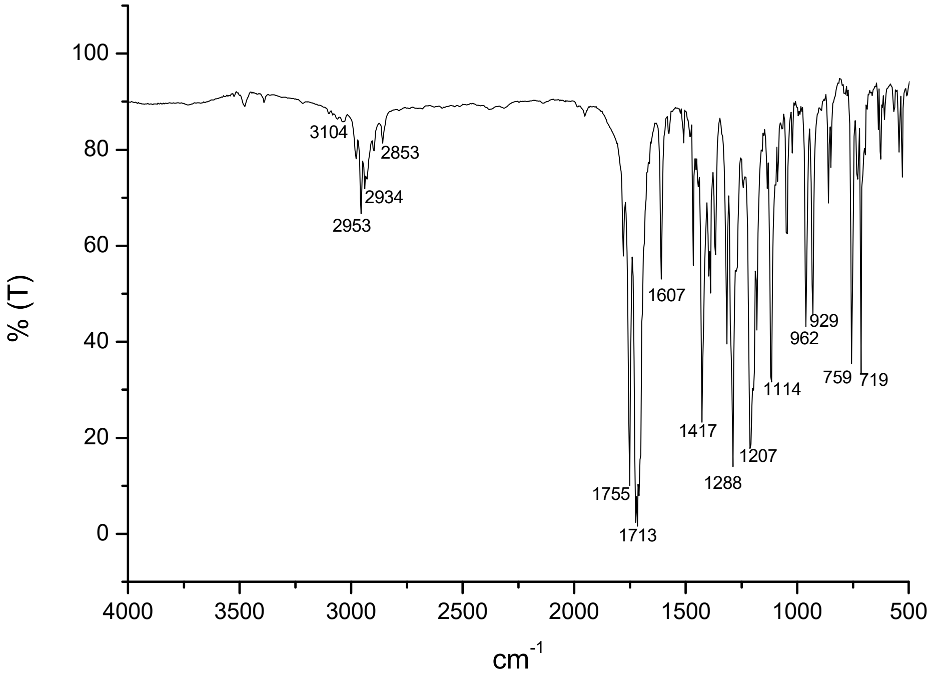

Figure S23. IR spectrum (KBr) of compound **4b**.

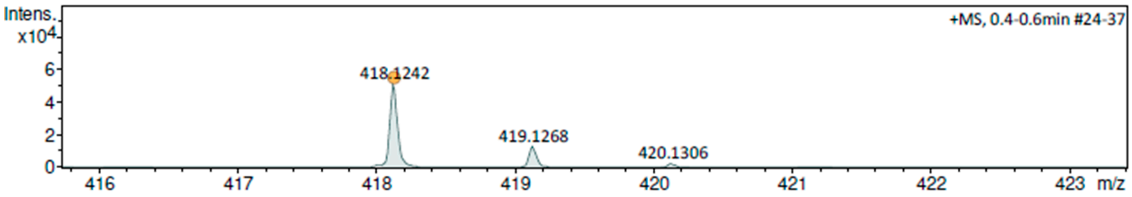

Meas. m/z	#	Ion Formula	m/z	err [ppm]	mSigma	# mSigma	Score	rdb	e⁻ Conf	N-Rule
418.1242	1	C22H21NNaO6	418.1261	4.5	2.7	1	100.00	12.5	even	ok

Figure S24. HRMS spectrum (ESI) of compound **4b**.

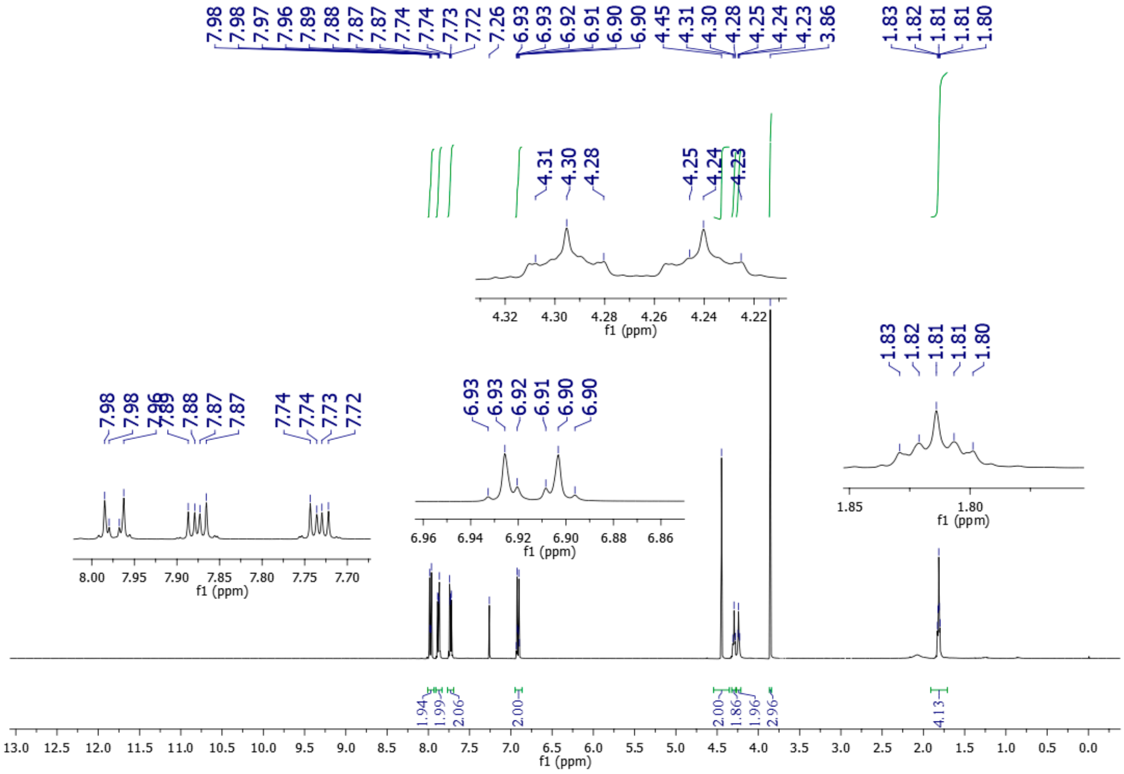

Figure S25. ^{1}H NMR spectrum (400 MHz, $CDCl_3$) of compound **4c**.

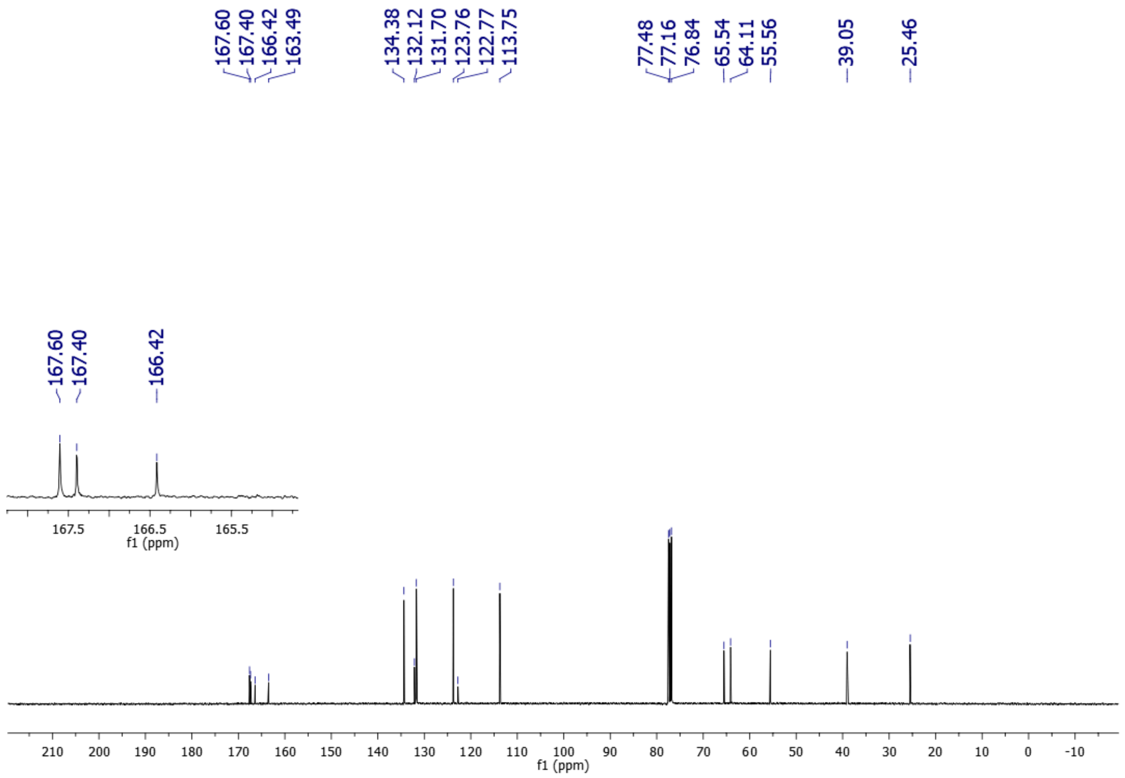

Figure S26. ^{13}C NMR spectrum (100 MHz, $CDCl_3$) of compound **4c**.

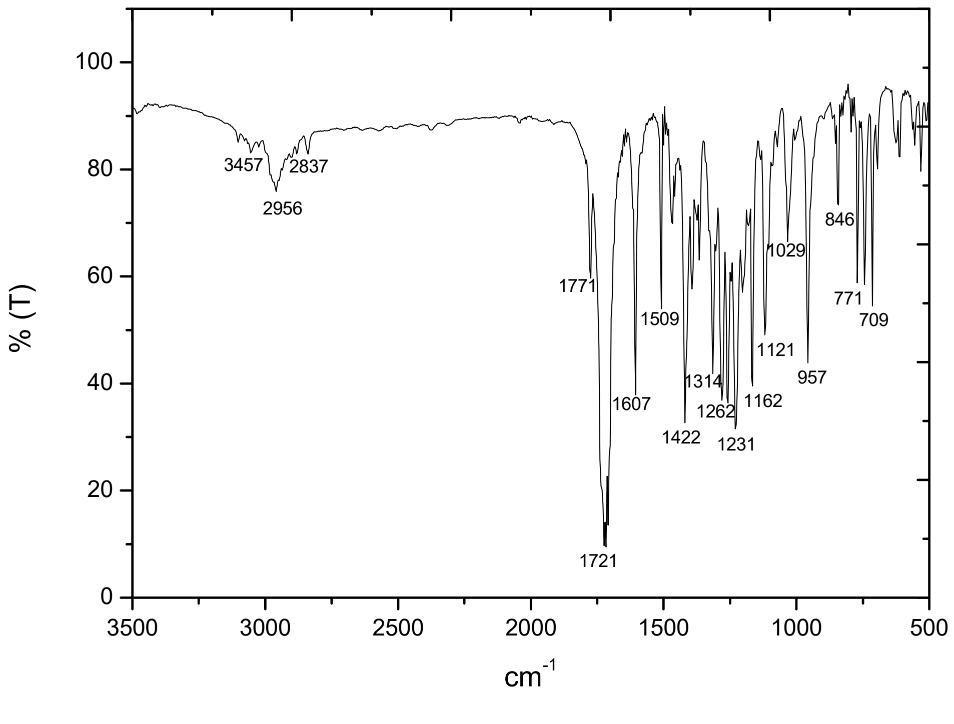

Figure S27. IR spectrum (KBr) of compound **4c**.

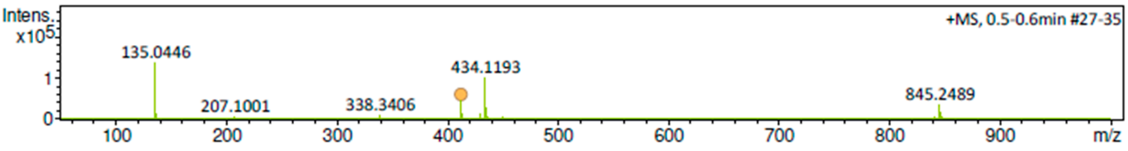

Meas. m/z	#	Ion Formula	m/z	err [ppm]	mSigma	# mSigma	Score	rdb	e⁻ Conf	N-Rule
412.1371	1	C22H22NO7	412.1391	4.8	1.3	1	100.00	12.5	even	ok

Figure S28. HRMS spectrum (ESI) of compound **4c**.

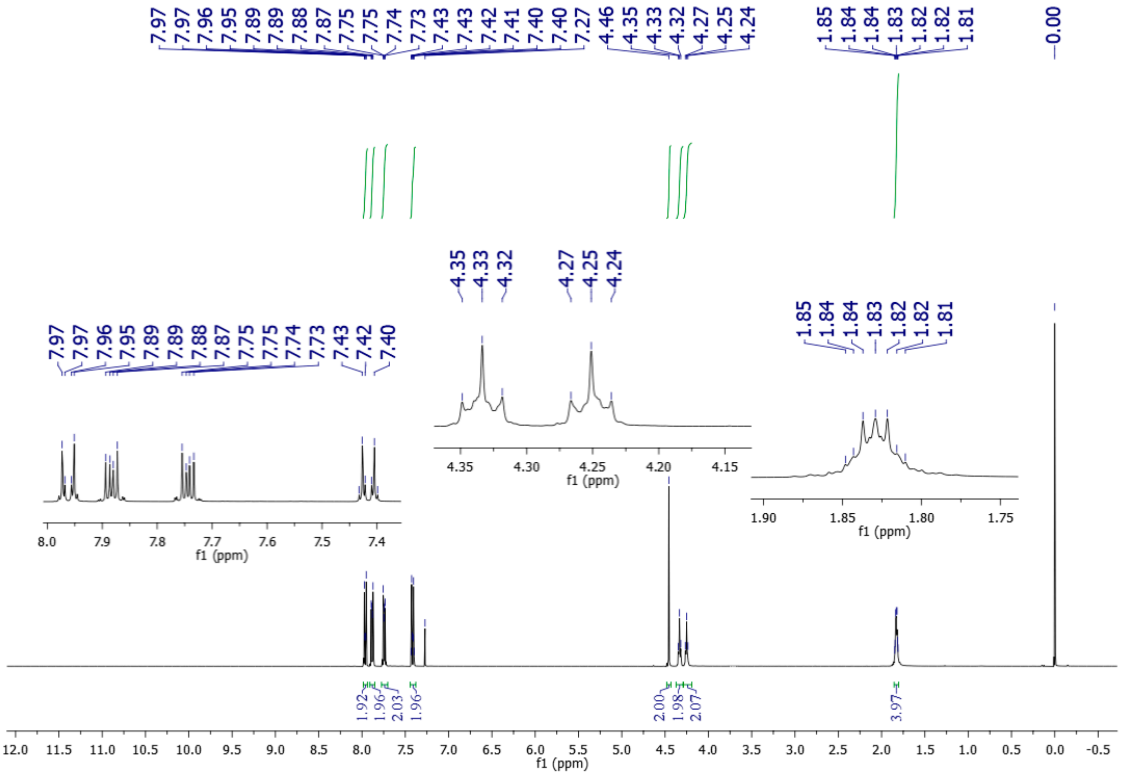

Figure S29. ^{1}H NMR spectrum (400 MHz, $CDCl_3$) of compound **4d**.

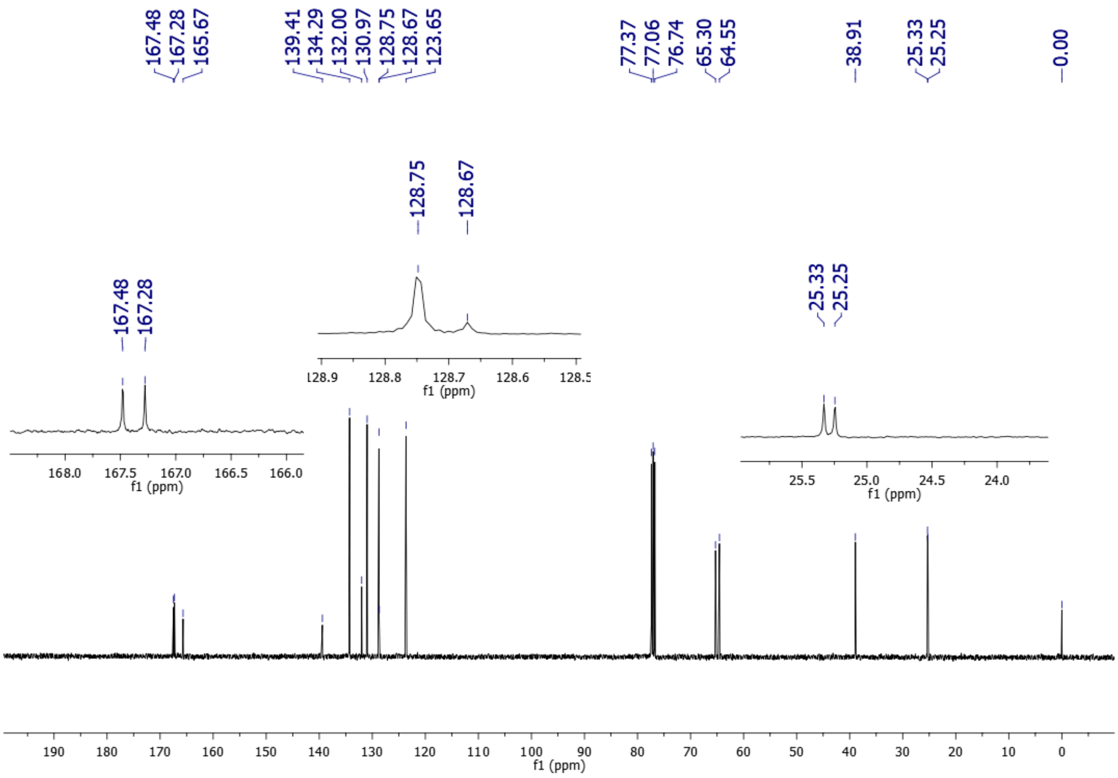

Figure S30. ^{13}C NMR spectrum (100 MHz, $CDCl_3$) of compound **4d**.

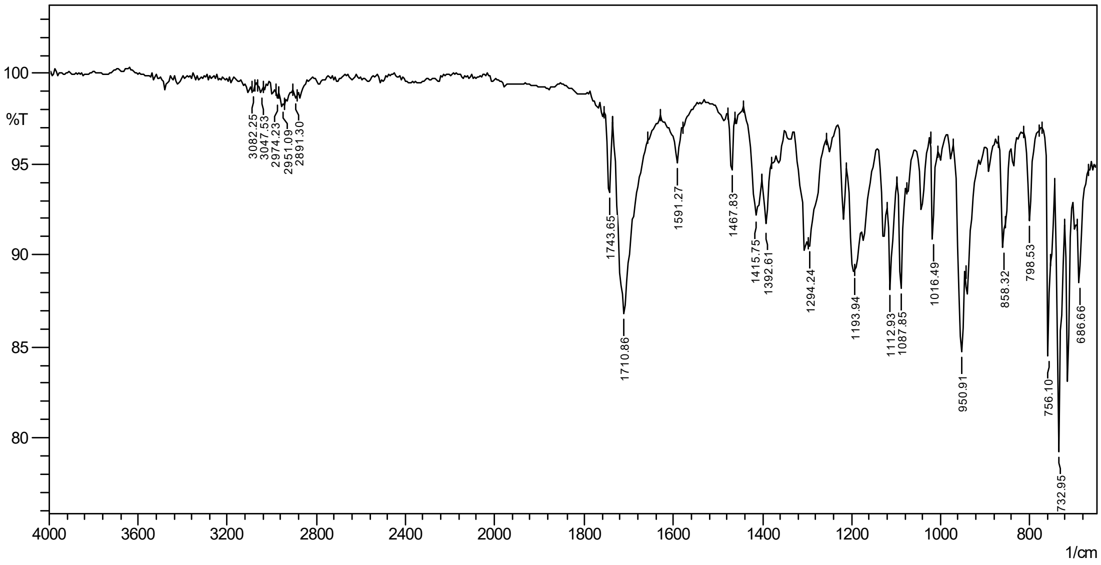

Figure S31. IR spectrum (ATR) of compound **4d**.

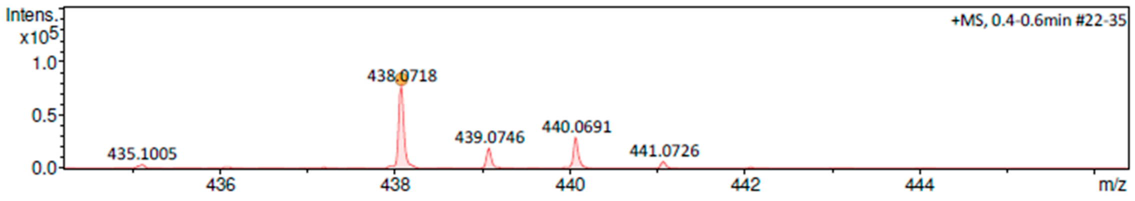

Meas. m/z	#	Ion Formula	m/z	err [ppm]	mSigma	# mSigma	Score	rdb	e⁻ Conf	N-Rule
438.0718	1	C21H18ClNNaO6	438.0715	-0.8	5.9	1	100.00	12.5	even	ok

Figure S32. HRMS spectrum (ESI) of compound **4d**.

Table S1. Estimated median lethal concentration (LC_{50}) for phthalimide and phthaloylglycine esters (**3b**, **3c**, **4a**, **4c**) on *Artemia salina* larvae using linear regression equation.

<table>
<tr><th>Compound</th><th>R</th><th>Graphical</th></tr>
<tr><td>3b</td><td>Me</td><td>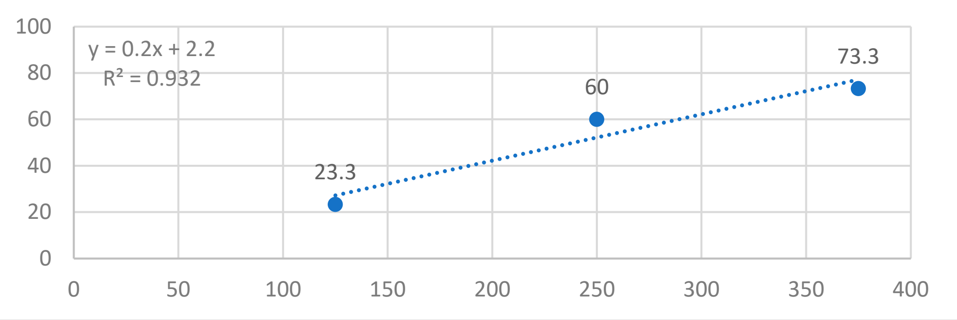
</td></tr>
<tr><td>3c</td><td>OMe</td><td>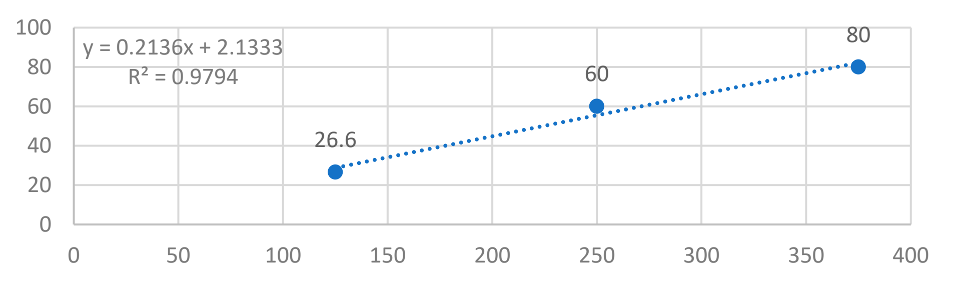
</td></tr>
<tr><td>4a</td><td>H</td><td>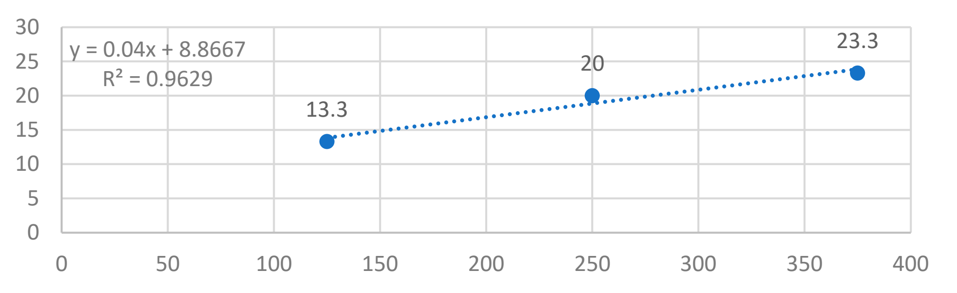
</td></tr>
<tr><td>4c</td><td>OMe</td><td>At concentrations of 125, 250 and 375 μg.mL^{-1}, any nauplius died.</td></tr>
</table>
